# Decoding the allosteric grammar of protein kinases: A dual‐stream framework integrating protein language models and energy landscape frustration analysis

**DOI:** 10.1002/pro.70714

**Published:** 2026-07-09

**Authors:** Will Gatlin, Max Ludwick, Lucas Turano, Brandon Foley, Kamila Riedlová, Vít Škrhák, Marian Novotný, David Hoksza, Gennady M. Verkhivker

**Affiliations:** ^1^ Keck Center for Science and Engineering, Department of Biological Sciences, Schmid College of Science and Technology Chapman University Orange California USA; ^2^ Department of Software Engineering, Faculty of Mathematics and Physics Charles University Prague Czech Republic; ^3^ Department of Cell Biology, Faculty of Science Charles University Prague Czech Republic; ^4^ Department of Biomedical and Pharmaceutical Sciences Chapman University School of Pharmacy Irvine California USA; ^5^ Department of Pharmacology, Skaggs School of Pharmacy and Pharmaceutical Sciences University of California San Diego La Jolla California USA

**Keywords:** ABL kinase, allosteric regulation, energy landscapes, local frustration, protein kinases, protein language models, structural bioinformatics

## Abstract

The spatial and energetic encoding of allosteric regulatory sites remains a major challenge in structural biology, frequently representing a “blind spot” for sequence‐based artificial intelligence (AI) models. We present a protein language model (PLM)‐guided approach complemented by the energy landscape frustration analysis as a dual‐stream framework to investigate the relationship between AI prediction of binding sites and biophysical organization of regulatory pockets across the human kinome. By probing a fine‐tuned residue‐level PLM classifier across 453 kinase structures, a clear performance gap is discovered between highly predictable orthosteric pockets (Types I, I.5, and II) and poorly resolved distal allosteric sites (Type IV). Rather than attempting to interpret this blind spot through internal AI attributions alone, we use independent local frustration profiles to analyze the underlying physics of these sites. We determine that the detectability of orthosteric and allosteric binding sites reflects their energetic embedding within the protein energy landscape. Orthosteric catalytic sites reside within minimally frustrated, optimized energetic regions that are consistently detected with high confidence. In contrast, allosteric sites are enriched in neutrally frustrated zones, producing diffuse and context‐dependent predictions. We demonstrate that this neutral frustration of functional regions acts as a biophysical lubricant, facilitating the conformational plasticity required for regulatory transitions while simultaneously eroding the coevolutionary signals exploited by PLMs. Atomic‐resolution analysis of abelson murine leukemia (ABL) kinase spanning multiple conformational states and complexes bound to diverse ligands provides mechanistic validation of this principle. The myristoyl allosteric pocket in ABL remains neutrally frustrated across complexes with physiological ligands, chemically diverse modulators, from allosteric inhibitors to activators, and conformations engaged with SH2–SH3 regulatory domains. We propose that allosteric sites are encoded in persistent neutrally frustrated regions optimized for context‐dependent regulatory modulation. This study reveals how the organization of the protein energy landscape shapes universal “allosteric grammar” and algorithmic detectability of regulatory binding sites.

## 1 INTRODUCTION

Allosteric interactions and communications in proteins are central to diverse biological mechanisms, and allosteric regulation has long been recognized as the “second secret of life.” (Astl et al., 2019; Changeux, 2012; Changeux & Edelstein, 2005; Dokholyan, 2016; Monod et al., 1965; Tsai & Nussinov, 2014; Wodak et al., 2019) Nonetheless, the diverse dynamic mechanisms that give rise to allosteric events continue to be fairly elusive and are often characterized at a phenomenological level, lacking a universal theoretical foundation that can explain the enormous diversity of these processes. This gap between experimental observation and mechanistic understanding is particularly acute at the intersection of allosteric regulation and the rapidly advancing fields of machine learning (ML) and artificial intelligence (AI)—a frontier where AI‐augmented integrative structural biology holds the promise of transforming how we discover and interpret the regulatory logic of allostery encoded in protein structures (Montserrat‐Canals et al., 2025; Nussinov, Zhang, Liu, & Jang, 2022). Recent years have witnessed the remarkable growth of data‐intensive experimental high‐throughput technologies and AI/ML tools aiming for atomistic‐level interrogation, characterization, and engineering of protein functions and mechanisms. Of particular interest for rapidly evolving AI tools in biology is accurate prediction of diverse regulatory allosteric sites in protein structures and dissecting biophysical principles that govern how certain regions of proteins emerge as hubs of regulatory control while others remain structurally inert.

In this work, we approach this longstanding question from a new perspective: rather than using AI solely as a predictive tool, we introduce and employ an integrated AI‐based framework complemented by energy landscape analysis as a diagnostic instrument to interrogate whether the predictive behavior of modern AI tools and particularly protein language model (PLM) can reveal the physical principles that encode the diversity of allosteric regulatory sites within protein structures.

Recent advances in large‐scale mutational profiling have begun to illuminate the global architecture of allosteric regulation. Pioneering studies from Lehner and colleagues used deep mutational scanning (DMS) and thermodynamic modeling to systematically map the energetic consequences of mutations across protein interaction domains (Beltran et al., 2026; Escobedo et al., 2025; Faure et al., 2022; Hidalgo‐Carcedo et al., 2026). These experiments revealed that allostery is not confined to a small set of specialized residues but instead emerges from distributed networks of interactions spanning entire protein domains. Importantly, these studies showed that allosteric landscapes exhibit a modular organization in which conserved structural cores are surrounded by more variable peripheral regions that diversify across homologs. They proposed a generalized mechanism where allostery evolves through the gain and loss of peripheral extensions to this conserved core, allowing for functional diversification while maintaining a fundamental allosteric mechanism (Hidalgo‐Carcedo et al., 2026). These illuminating findings suggest that regulatory potential may be encoded not simply in individual residues but in the broader energetic organization of the protein structure and the underlying energy landscape where rigid protein cores communicate with flexible regions to mediate context‐dependent regulation—a principle that directly resonates with the frustration‐based integrated AI framework we develop in the current investigation.

Protein kinases provide a particularly powerful system for exploring these ideas. Allosteric regulation has long been viewed as the hidden language of cellular signaling (Changeux & Edelstein, 2005) and yet biophysical grammar that encodes regulatory diversity at spatial and evolutionary levels remains elusive. As central regulators of cellular signaling, protein kinases are by far among the best‐characterized protein families both structurally and functionally, with thousands of experimental structures spanning multiple conformational states and ligand‐bound forms (Meharena et al., 2013; Taylor et al., 2012). The extensive structural and biochemical studies have revealed how functional elements of the kinase domains (KDs) reorganize during activation and inhibition, producing a rich mechanistic picture of kinase regulation (Ahuja et al., 2019; Taylor et al., 2021; Verkhivker, 2021). This wealth of structure‐functional data, combined with rigorous classification systems that distinguish orthosteric adenosine triphosphate (ATP)‐competitive inhibitors from proximal and distal allosteric modulators (Faezov & Dunbrack Jr., 2023; Gizzio et al., 2026; Modi & Dunbrack Jr., 2019, 2022) enables systematic comparison of different classes of binding sites and regulatory regions within a common structural framework. Moreover, protein kinases possess a remarkable diversity of regulatory interfaces distributed across their catalytic domains that reflects an evolutionary strategy in which signaling enzymes exploit multiple structural sites to fine‐tune activity and integrate diverse cellular signals, providing a rich landscape for investigating how allosteric sites are encoded (Laufkötter et al., 2021; Xerxa et al., 2023; Xerxa & Bajorath, 2023).

While experimental studies have revealed the richness of allosteric regulation, computational prediction of regulatory regions and particularly allosteric sites remains a major challenge. Deep learning (DL) approaches and PLMs trained on large protein sequence datasets (Lin et al., 2023; Madani et al., 2023; Schmirler et al., 2024; Sledzieski et al., 2024; Zhang et al., 2024) have achieved remarkable success in proteome‐wide functional site annotation, with particular strength in identifying conserved binding sites (Carbery et al., 2024; Gamouh et al., 2025; Rohulia et al., 2025; Sim et al., 2025). Structure‐based ML algorithms (Jian et al., 2016; Jiménez et al., 2017; Krivák & Hoksza, 2018; Polák et al., 2025) exploited geometric and physicochemical features to robustly identify canonical orthosteric ligand‐binding pockets in protein structures. Despite these advances, robust detection of functional allosteric sites remains challenging, as most methods are optimized for stable, evolutionarily conserved canonical binding pockets, while allosteric sites are often transient, weakly conserved, and sparsely represented in structural databases (Li et al., 2025). A recent study combined local binding geometry, coevolutionary information, and dynamic allostery into a multiparameter framework designed to identify potentially hidden allosteric sites within ensembles of protein structures bound to orthosteric ligands (La Sala et al., 2023). The emerging dichotomy in predictions of canonical orthosteric and regulatory allosteric binding sites is commonly attributed to limited training data or methodological inefficiencies. While these factors undoubtedly contribute to a markedly reduced prediction performance of PLMs and ML tools on allosteric sites, a more granular analysis from experimental studies suggests a deeper explanation. If regulatory sites occupy regions that are evolutionarily permissive and structurally adaptable—features necessary for accommodating diverse regulatory interactions—then they may lack the strong sequence conservation patterns that ML models rely upon. In this view, the apparent blind spot of AI algorithms may reflect the intrinsic evolutionary design of regulatory regions rather than a deficiency of current predictive methods.

Energy landscape theory provides a powerful framework for exploring this possibility (Ferreiro et al., 2007; Parra et al., 2016). Rather than viewing proteins as static structures, this perspective describes them as ensembles of conformational states organized on a multidimensional energy landscape whose topology governs folding, dynamics, and functional transitions. Local frustration analysis offers a quantitative approach for interrogating this landscape by evaluating how favorably individual interactions are optimized relative to alternative configurations (Chen et al., 2020; Ferreiro et al., 2007; Parra et al., 2016). Evolution tends to minimize frustration in regions essential for structural stability, creating strongly funneled energetic basins that ensure robust folding. In contrast, regions involved in binding, signaling, or conformational switching often retain higher levels of frustration, enabling conformational flexibility and facilitating functional transitions (Chen et al., 2020). Across many protein families, minimally frustrated residues cluster in conserved structural cores, whereas frustrated or neutrally frustrated interactions often appear at ligand‐binding pockets, protein–protein interfaces, and conformational switch regions (Chen et al., 2020; Freiberger et al., 2021; Gianni et al., 2021; Parra et al., 2025). The frustration patterns have been linked to evolutionary conservation, conformational dynamics, and allosteric communication pathways (Freiberger et al., 2021; Gianni et al., 2021; Parra et al., 2025). Combined, these observations raise the possibility that the energetic embedding of binding pockets within the protein landscape may determine both their regulatory behavior and their evolutionary signatures.

In this study, we integrate PLM predictions of orthosteric and allosteric binding sites with the energy landscape analysis into a synergistic AI framework to probe protein allostery and interrogate the organization of regulatory sites in protein kinases. Rather than using PLMs solely as a predictive tool, we complement AI results with independent physics‐centric landscape analysis which combined form a diagnostic instrument and biophysical lens to probe the energetic embeddings of diverse binding sites and regulatory regions in the global architecture of protein kinase structures. Our investigation unfolds in three integrated steps. First, we systematically characterize the differential detectability of orthosteric and experimentally characterized allosteric sites using PLM predictions across a curated dataset of 453 human kinases from the KinCoRe database (Modi & Dunbrack Jr., 2022). This analysis establishes that orthosteric and allosteric sites occupy distinct regimes of algorithmic visibility—a pattern that raises questions about interplay and balance between methodological limitations and biophysical origins. Second, we introduce local frustration analysis as a biophysical interpretability layer, testing whether the observed performance patterns correspond to underlying differences in how these sites are embedded within the energy landscape.

By comparing frustration signatures across site classes and mapping their spatial distribution, we ask whether orthosteric and allosteric sites exhibit fundamentally different energetic organization. Third, we examine these principles at atomic resolution using the ABL kinase system, which provides a uniquely well‐characterized example of diverse allosteric regulatory hubs. By analyzing a comprehensive set of ABL structures—including complexes with orthosteric inhibitors, allosteric modulators of opposing function, and assemblies with regulatory domains—we investigate how conformational and mutational frustration patterns manifest across different regulatory contexts and examine how neutral frustration persists across conformational states and enables functional versatility of the diverse allosteric sites in the human kinome.

Our results reveal a consistent organizing principle: orthosteric catalytic sites are embedded within minimally frustrated regions of the energy landscape that are strongly constrained by evolution and therefore readily detected by AI models, whereas allosteric sites are encoded within neutrally frustrated zones of the energy landscape which underlie their regulatory versatility and limited detectability by AI models. More broadly, these findings demonstrate how integration of PLM predictions and landscape‐based frustration analysis can be used not only to identify functional regions and binding sites but serve as diagnostic probes of the physical principles governing their evolutionary design, transforming AI tools into a powerful instrument for interrogating protein function.

## 2 RESULTS

### 2.1 An integrative dual‐stream framework: PLM as evolutionary probe and frustration analysis as physical interpretability layer

**FIGURE 1:**
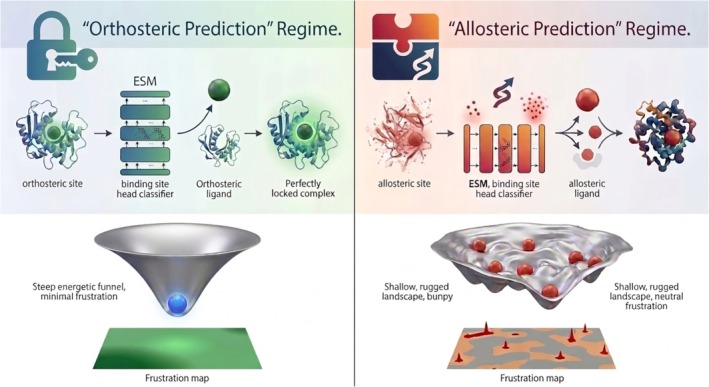
A diagnostic framework linking protein language model (PLM) predictions to energy landscape organization. (Left panel) Orthosteric prediction regime: PLM encounters an orthosteric pocket characterized by a steep energetic funnel with minimal frustration. The PLM detects this site with high confidence; the frustration map shows a concentrated region of minimal frustration (green) at the binding interface. (Right panel) Allosteric prediction regime: the same model encounters a dynamic allosteric pocket with a shallow, rugged landscape dominated by neutral frustration. The PLM produces diffuse, low‐confidence predictions; the frustration map shows a neutrally frustrated pocket interior (red) with surrounding frustrated switch points (magenta). Bottom panels illustrate energy landscape schematics: orthosteric sites occupy deep, funneled basins that generate strong coevolutionary signals, while allosteric sites reside in shallow landscapes with multiple minima, enabling regulatory versatility at the cost of sequence‐based detectability.

To understand why certain binding sites are readily detectable while others remain elusive, we proposed a theoretical formulation that reframes the question from “how well do models predict?” to “what do prediction patterns reveal about how evolution encodes functional sites within protein energy landscapes?” The allosteric blind spot is typically attributed to data sparsity or algorithmic limitations. However, an alternative possibility is that the limited detectability of allosteric sites reflects intrinsic properties of their evolutionary and energetic design. Distinguishing between these explanations requires an integrated strategy that can connect algorithmic behavior to underlying biophysical principles. To address this, we introduce an AI framework that treats computational PLM predictions of binding sites not as endpoints but as diagnostic probes of the protein energy landscape organization (Figure 1). The first stream employs PLM as an unbiased evolutionary probe. When such a model encounters a binding site, its prediction confidence reports on how strongly that site is constrained by evolution—a quantitative readout of sequence‐based detectability. The second stream employs the landscape‐based local frustration analysis. This framework evaluates how favorably each native residue–residue interaction is optimized relative to alternative configurations, classifying residues into three energetic classes: minimally frustrated (interactions strongly favored over alternatives, reflecting evolutionary optimization), neutrally frustrated (interactions neither strongly favored nor disfavored, permitting conformational plasticity), and highly frustrated (interactions less favorable than alternatives and storing local strain) (Chen et al., 2020; Ferreiro et al., 2007; Freiberger et al., 2021; Gianni et al., 2021; Parra et al., 2016, 2025). This provides a physically grounded language for describing how different regions of a protein are embedded within the energy landscape. The critical insight emerges from overlaying these two streams (Figure S1). If prediction performance correlates with frustration signatures, then PLM behavior becomes interpretable through protein physics rather than remaining an opaque statistical output. Specifically, we hypothesized that canonical orthosteric sites—evolutionarily locked, catalytically essential pockets—would reside in minimally frustrated basins where native interactions are strongly favored and evolutionary conservation is high, generating robust coevolutionary signals reliably detectable by PLMs. On the other hand, allosteric sites, designed for conformational plasticity and context‐dependent function, would occupy shallow, rugged regions of the energy landscape dominated by neutral frustration. This neutrality permits sequence drift and structural adaptability, enabling regulatory versatility, but also erodes the statistical signals that sequence‐based models require. When a PLM encounters such a site, its predictions should become more diffuse, low‐confidence, and structure‐dependent—a regime where the pocket is only weakly detectable in most conformations.

### 2.2 Structural diversity of kinase allosteric sites defines a natural testbed for probing allostery and binding‐site detectability

**FIGURE 2:**
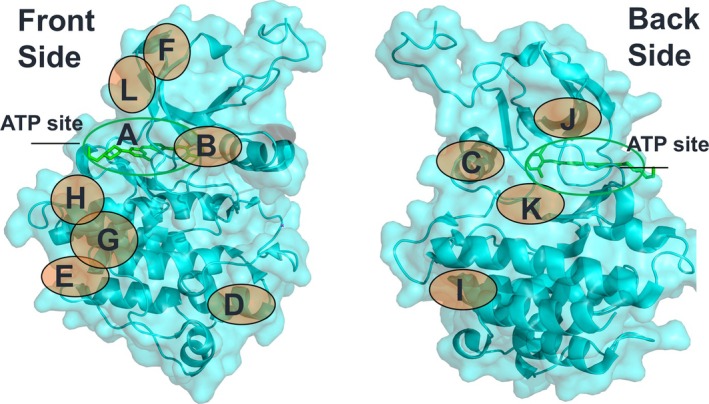
Structural diversity of allosteric binding sites across the kinase fold. The 12 canonical allosteric binding regions, compiled from a survey of 262 allosteric kinase ligands (Laufkötter et al., 2021; Xerxa et al., 2023) are mapped onto a representative ABL kinase structure (Protein Data Bank 2GQG). Allosteric modulators target a wide range of locations, including the myristoyl pocket in the C‐lobe (Type IV), the back pocket adjacent to the ATP site (Type III), and multiple surface grooves. The orthosteric Type I Dasatinib bound in the ATP site is shown in green sticks.

To investigate how evolutionary constraints shape binding site detectability, we used protein kinase family that simultaneously satisfies three criteria: a conserved catalytic architecture, extensive structural coverage across functional states, and a diverse repertoire of regulatory allosteric pockets (Figure 2) (Ahuja et al., 2019; Meharena et al., 2013; Taylor et al., 2012, 2021; Verkhivker, 2021). The KinCoRe classification system provides standardized annotation of kinase conformational states and inhibitor binding modes (Faezov & Dunbrack Jr., 2023; Gizzio et al., 2026; Modi & Dunbrack Jr., 2019, 2022). Based on specific structural markers—the aspartate‐phenylalanine‐glycine (DFG) motif (Asp‐Phe‐Gly) and the regulatory αC‐helix position—this system categorizes inhibitors into five mechanistic classes (Figure S2). Types I, I.5, and II inhibitors occupy the orthosteric ATP‐binding cleft but capture different conformational states: Type I binds the active DFG‐in conformation, Type I.5 represents a transitional state with the αC‐helix shifted outward, and Type II binds the inactive DFG‐out conformation. Type III inhibitors are proximal allosteric modulators that bind pockets adjacent to the ATP site but do not enter the cleft itself, while Type IV inhibitors are distal allosteric modulators that bind sites far from the catalytic machinery (Figure S2). The evaluation dataset derived from the KinCoRe database (Faezov & Dunbrack Jr., 2023; Gizzio et al., 2026; Modi & Dunbrack Jr., 2019, 2022) provided initially a total of 9901 kinase–ligand complexes (spanning 453 human kinases) (Table 1). The dataset includes all five classes of inhibitor binding. The full dataset encompasses 10,301 complexes spanning 453 human kinases, with orthosteric sites heavily represented (7604 Type I complexes) and far sparser representation for distant allosteric sites (675 Type IV complexes).

**TABLE 1:** Composition of the kinase dataset derived from KinCoRe.

Inhibitor class	Binding mode	KinCoRe (full)	KinCoRe‐UNQ
Type I	ATP‐competitive (DFG‐in, C‐in)	7604	407
Type I.5	ATP‐competitive (C‐out extension)	775	117
Type II	ATP‐competitive (DFG‐out)	553	65
Type III	Proximal allosteric	294	30
Type IV (ALLO)	Distal allosteric	675	89
Total	‐	9901	708

To visualize the diversity of allosteric sites across the kinase fold, we mapped the 12 canonical allosteric binding regions compiled from a recent survey of allosteric kinase ligands (Laufkötter et al., 2021; Xerxa et al., 2023) (Figure 2). This structural atlas reveals two important features simultaneously: significant diversity in allosteric site locations, yet recurrent patterns across the kinase family. Allosteric modulators target a wide range of locations, including the well‐characterized myristoyl pocket in the C‐lobe (Type IV), the back pocket adjacent to the ATP site (Type III), and several less‐conserved surface grooves. This spatial diversity reflects an evolutionary strategy of exploiting multiple regulatory interfaces for fine‐tuned control. Despite this diversity, allosteric sites consistently occupy regions peripheral to the conserved catalytic core. Only three of the binding sites of Type IV inhibitors represented deep pockets (D, E, and H) (Laufkötter et al., 2021; Xerxa et al., 2023). Of special importance is deep pocket E in the C‐terminal lobe that corresponds to the myristoyl pocket in ABL kinase (Figure 2). Because structural datasets are inherently imbalanced with certain kinases being overrepresented (Table 2), we also constructed a non‐redundant subset KinCoRe‐UNQ where each protein (identified by its UniProt accession) is included only once. For each UniProt record, we randomly selected one structure from the original dataset yielding 708 complexes which enables the evaluation of AI predictions by removing bias for structural over‐representation.

**TABLE 2:** Grouped categories used for protein language model performance benchmarking.

Site category	Classes included	Count	Fraction (%)
Orthosteric	Types I, I.5, II	8932	90.2%
Allosteric (proximal)	Type III	294	3.0%
Allosteric (distal/ALLO)	Type IV	675	6.8%

Among kinase systems, ABL kinase provides a particularly informative experimental platform for probing and decoding allosteric site diversity using our integrated AI framework (de Buhr & Gräter, 2023; Nagar et al., 2003, 2006; Panjarian et al., 2013). The extensive structural characterization of ABL complexes, including a spectrum of active and inactive apo states (Saleh et al., 2017; Xie et al., 2020), regulatory domain assemblies with the SH2–SH3 module (Lamontanara et al., 2014; Paladini et al., 2024; Skora et al., 2013; Sonti et al., 2018), and numerous ligand‐bound conformations (Astl & Verkhivker, 2019; Manning et al., 2002; Metz et al., 2018; Moret et al., 2020; Nussinov, Zhang, Maloney, et al., 2022; Schoepfer et al., 2018; Wylie et al., 2017; Xie et al., 2022; Yang et al., 2011; Zhang et al., 2010) provides an ideal system for examining how energetic organization shapes allosteric ligand recognition. Using the ABL1 kinase as a representative architecture, we highlight the complex multi‐domain organization that couples the catalytic KD to regulatory SH3 and SH2 modules (Figure S3A–C). The regulatory myristoyl pocket in ABL (Figure 2) exemplifies the functional versatility of allosteric sites by functioning not as a simple regulatory switch but as a versatile allosteric hub capable of accommodating chemically diverse ligands and producing distinct functional outcomes (Figure S3). Structural studies showed that this allosteric binding pocket exists in both ligand‐free apo structures and kinase complexes where some ligands can induce a significant conformational change in the pocket that triggers autoinhibition, while allosteric activators stabilize the native conformation (Laufkötter et al., 2021; Xerxa et al., 2023). Our previous computational studies have also mapped the intricate allosteric networks in ABL kinase, identifying communication pathways that connect the peripheral myristoyl‐binding site to the orthosteric ATP pocket (Figure S3D,E). These findings provide a structural foundation for exploring how “rigid” functional motifs (minimally frustrated regions) are coupled with structurally adaptable peripheral regions (neutrally frustrated zones)—a relationship recently highlighted in broader proteomic studies (Beltran et al., 2026; Escobedo et al., 2025; Faure et al., 2022; Hidalgo‐Carcedo et al., 2026). Taken together, the structural diversity of kinase regulatory pockets, combined with the conserved architecture of the catalytic site and the detailed mechanistic understanding of ABL allostery, creates a natural experimental landscape and a high‐resolution testbed for using PLM predictions to probe how evolutionary constraints influence binding site detectability.

### 2.3 Hierarchy of binding site detectability across the human kinome: PLM performance as a diagnostic probe of the allosteric blind spot

**FIGURE 3:**
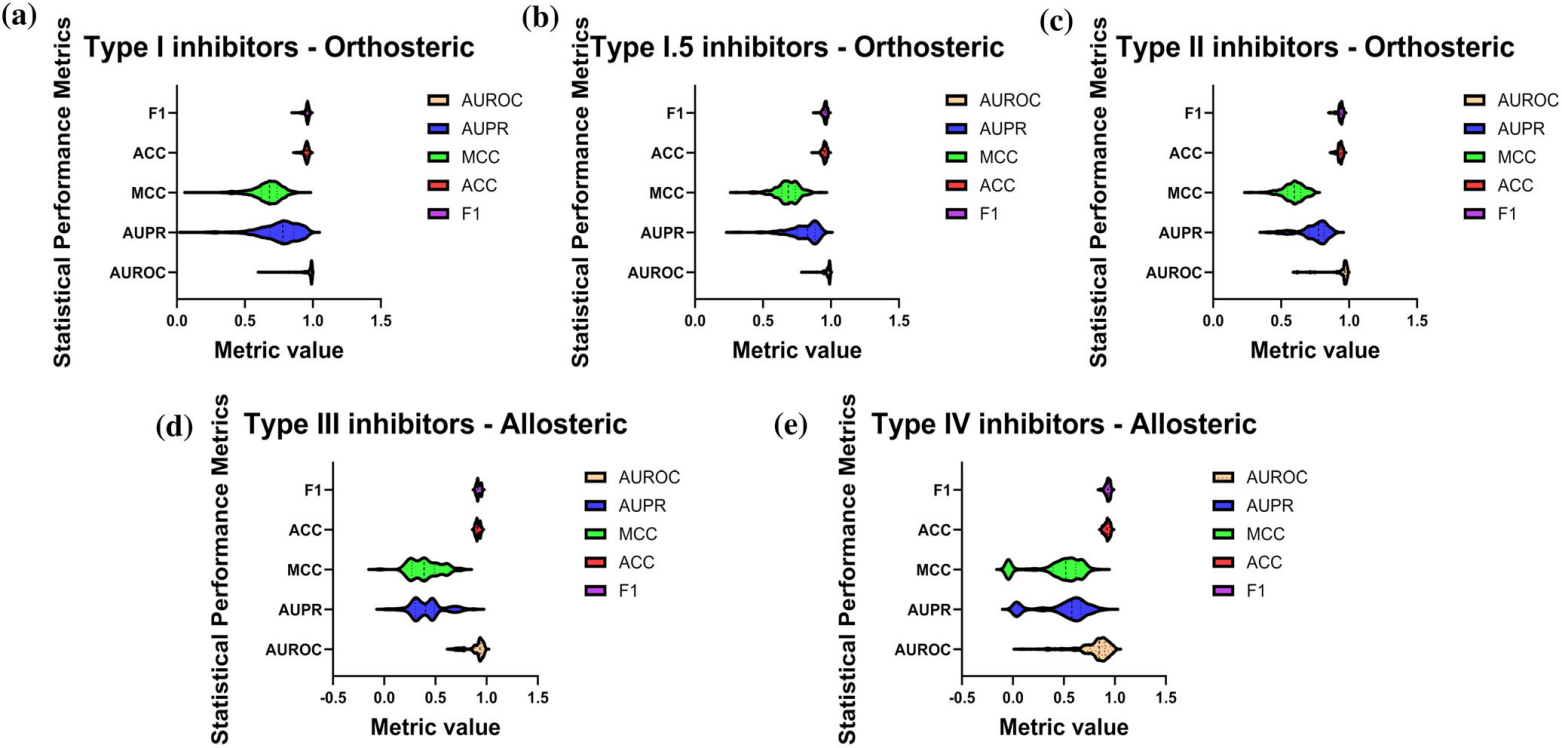
Protein language model (PLM) analysis across orthosteric and allosteric kinase sites reveals a systematic predictability gap. Violin plots display the distribution of five classification metrics (area under the receiver operating characteristic curve [AUROC], area under the precision–recall curve [AUPR], Matthews correlation coefficient [MCC], Accuracy, and F1 score) for PLM predictions across five inhibitor classes from the KinCoRe dataset. Each violin represents the density of per‐structure performance values, with overlaid box plots indicating median and interquartile range. The baseline sequence model was trained on the independent LIGYSIS_SI30 dataset (*N* = 2949 sequences). Performance distributions are reported for both the full testing repository (*N* = 9901 complexes) and the non‐redundant testing dataset (*N* = 708 complexes). The specific sample size (*n*) breakdown per inhibitor/binding‐site class within the full repository and the non‐redundant test set (full/non‐redundant) is as follows: Type I (orthosteric): *N* = 7604/*n* = 407 structures; Type I.5 (orthosteric/gatekeeper): *N* = 775/*n* = 117 structures; Type II (orthosteric/DFG‐out): *N* = 553/*n* = 65 structures; Type III (proximal allosteric): *N* = 294/*n* = 30 structures; Type IV (distal allosteric): *N* = 675/*n* = 89 structures. (a–c) Orthosteric ATP‐competitive inhibitors: Type I (a), Type I.5 (b), and Type II (c) inhibitors exhibit tight distributions with median values near 1.0 and minimal interquartile ranges (IQR ≈0.02–0.03), indicating universal detectability across all kinase structures. (d, e) Allosteric inhibitors: Type III (d) and Type IV (e) allosteric modulators show dramatically broader, lower‐performance distributions. Type III sites display moderate‐to‐broad distributions (median ≈0.36 for AUPR), reflecting structure‐dependent variability, while Type IV sites exhibit extreme distribution spread (median AUPR ≈0.08), indicating statistical invisibility in most conformations. As the PLM outputs for each residue a probability $p_{i}\in \left[0,1\right]$ that the residue belongs to a ligand‐proximal site, we threshold the probabilities to obtain binary predictions. In the PLM fixed setting, the probabilities were binarized using a single threshold t=0.75. Raw probabilities were retained for threshold‐free analyses using AUROC and AUPR. The classification metrics (MCC, Accuracy, and F1) were calculated using a consistent fixed confidence threshold to ensure uniformity across diverse structural contexts. ACC, Activation loop conformation.

To systematically assess binding‐site detectability, we adapted and employed a previously developed fine‐tuned PLM for residue‐level binding site prediction (Riedlová et al., 2026). This implementation leveraged the pretrained evolutionary scale modeling (ESM)2‐650M model (Lin et al., 2023) as a baseline and was optimized on the human subset of the LIGYSIS dataset (Utgés et al., 2025; Utgés & Barton, 2024). The masked language modeling head was replaced with a specialized token‐level classification layer that maps each residue's 1280‐dimensional latent representation to a calibrated probability of ligand involvement (Riedlová et al., 2026) (Figure S1). Using this fine‐tuned PLM as an unbiased algorithmic probe, we evaluated its predictions of binding sites across all 453 human kinases in the KinCoRe dataset (Modi & Dunbrack Jr., 2022) which provides rigorous geometry‐based classification of inhibitor‐bound structures into five mechanistic classes: Types I, I.5, and II (orthosteric ATP‐competitive inhibitors) and Types III and IV (proximal and distal allosteric modulators) (Riedlová et al., 2026). For each structure, we computed standard metrics area under the receiver operating characteristic curve (AUROC), area under the precision–recall curve (AUPR), MCC, accuracy, and F1 score to quantify the PLM ability to identify ligand‐binding residues. The resulting distributions are shown in Figure 3.

Noteworthy, while our initial methodological study (Riedlová et al., 2026) introduced the fine‐tuned PLM and benchmarked the predictive PLM performance and algorithmic parameters, in the current study we deploy these metrics as diagnostic signatures and quantitative readouts revealing how the protein energy landscape can dictate the encoding of orthosteric sites versus allosteric binding pockets in protein kinases with special emphasis on atomistic‐level analysis of the ABL kinase.

The AUROC metric, which captures global ranking ability independent of decision thresholds, distinguishes the three classes. Orthosteric Types I, I.5, and II sites exhibit remarkably narrow and tall distributions, with medians between 0.94 and 0.98 and interquartile ranges of only 0.02–0.03. This means that almost every individual structure was predicted with high precision (high density at high scores), reflected in tight distributions with a consistent median. This exceptional consistency indicates that orthosteric sites are universally rankable across all kinase structures as the PLM distinguishes them from background regardless of conformational state (Figure 3a–c).

For proximal Type III allosteric sites, the AUROC distribution broadens considerably (median ≈0.91, IQR ≈0.06–0.08) (Figure 3d,e). This widening reflects a pronounced structure‐dependent variability whereby some protein kinase conformations allow near‐orthosteric ranking, while the majority of predictions yield substantially lower values. The distribution is no longer a tall peak but a wider mountain, signaling that detectability is contingent on structural context rather than universal. Distal Type IV allosteric sites display the most extreme pattern characterized by an extremely broad, flattened distribution (median ≈0.68, IQR ≈0.12–0.15) with many structures yielding near‐random ranking and a long lower tail extending to 0.5 (Figure 3e). This spread reveals that Type IV allosteric binding sites are statistically invisible in most conformations, with detectable signal present only in a subset of structures.

The AUPR metric, which measures operational precision under extreme class imbalance, exposes the true extent of the allosteric blind spot. Orthosteric sites maintain usable precision across most structures, with moderately broad but right‐skewed distributions (median AUPR 0.63–0.75, IQR ≈0.08–0.10) (Figure 3a–c). The skew reflects class imbalance rather than site ambiguity—some structures yield slightly lower recall, but precision remains robust. Type III proximal allosteric sites show broader AUPR distributions (median ≈0.36, IQR ≈0.15–0.20), spanning from approximately 0.10 to 0.60 (Figure 3d). This extreme width indicates that precision is highly structure‐dependent where only certain conformations can reveal the site with moderate precision. For Type IV sites, AUPR distributions are compressed near‐zero, with medians of only 0.08 and spreads that place most structures near the random baseline of 0.02–0.08 (Figure 3e). The characteristic pattern of high AUROC coupled with vanishing AUPR for Type IV sites reveals that while allosteric residues may be globally rankable, they lack the discriminative evolutionary signatures required for confident detection, reflecting their design for conformational plasticity rather than conserved recognition.

MCC metric which is the most informative measure for imbalanced data when using a fixed confidence threshold confirms and refines this hierarchy. Orthosteric sites demonstrate tight, high distributions (median MCC 0.58–0.69, IQR ≈0.05–0.07), with a sharp peak in the 0.6–0.7 range and very few structures falling below 0.4 (Figure 3a–c). This consistency reflects high‐quality classification across kinase structures. Type III sites exhibit broader, lower distributions (median MCC 0.38–0.45, IQR ≈0.10–0.12) (Figure 3d). While some structures still achieve MCC above 0.5, many fall to 0.2–0.3, with the distribution spanning from near‐zero to moderately positive—a direct reflection of conformational ambiguity. Type IV sites collapse toward zero (median MCC <0.15, IQR ≈0.12–0.15), with most mass lying between −0.1 and +0.2 and only a long tail extending to perhaps 0.3–0.4 for rare outliers (Figure 3e). Many structures yield MCC values indistinguishable from random, confirming statistical invisibility. Accuracy, which is influenced by the large number of True Negatives (TN), shows only subtle differentiation: orthosteric sites achieve very high median accuracy (0.94–0.96) with extremely tight distributions (IQR ≈0.01–0.02) (Figure 3a–c); Type III sites show a slight downward shift and increased spread (median 0.88–0.92, IQR ≈0.03–0.04) (Figure 3d); and Type IV sites exhibit a longer lower tail (median 0.86–0.90, IQR ≈0.04–0.06), indicating that false positives become numerous enough to degrade accuracy in some structures (Figure 3e).

The distribution statistics for kinome‐wide performance metrics demonstrated high‐ranking performance across orthosteric sites, while showing a significant performance decay in Type IV allosteric regions (Tables S1–S5). While AUROC remains relatively high for allosteric sites, the precipitous drop in AUPR and MCC confirms that these sites are statistically “blind” in most Type IV allosteric sites as shown in Tables S2 and S3. While accuracy alone does not reveal the allosteric blind spot as it is dominated by TN, the pattern remains consistent. The weighted F1 score, sensitive to positive‐class performance, captures the precision collapse more vividly: orthosteric sites maintain high F1 across structures (median 0.94–0.96, IQR ≈0.03–0.05); Type III sites show very broad distributions (median 0.86–0.91, IQR ≈0.08–0.10); and Type IV sites, despite modestly high medians (0.85–0.89), exhibit a lower tail extending below 0.7 for many structures (Figure 3).

To summarize, our results show that orthosteric sites are universally detectable in PLM predictions. In contrast, the broader detectability of proximal Type III allosteric sites depends on the structural context while distal Type IV allosteric sites often represent a blind spot for PLM predictions in most kinase structures.

### 2.4 The allosteric blind spot is robust to alternative pocket boundaries and dynamic loop masking

A potential source of ambiguity in sequence‐based functional site benchmarking is the mapping procedure used to translate a three‐dimensional binding pocket into discrete residue‐level annotations. Because allosteric pockets are inherently dynamic, sensitive to conformational state transitions, and frequently composed of spatially discontinuous sequence segments, we conducted a comprehensive robustness analysis to verify whether our findings are invariant or sensitive to alternative definitions of pocket boundaries, contact distance criteria, and inclusion or masking of flexible loop regions. First, we evaluated the impact of systematically altering the heavy‐atom distance threshold used to establish ground‐truth residue labels, varying the cutoff from a highly restrictive to a highly permissive (Table S6). This table presents the macro‐averaged performance metrics (median and interquartile range) evaluated across human kinase structures, categorized by inhibitor class, demonstrating that the predictive hierarchy is completely stable across different pocket boundary definitions. If the allosteric blind spot were simply an artifact of the baseline cutoff, expanding or restricting the boundary would be expected to narrow the performance gap between orthosteric and allosteric sites. By altering the distance cutoff used to define ground‐truth residue labels from 3.5 to 5.0 Å, in 0.5 Å increments (Table S6). We found that for orthosteric sites (Types I, I.5, and II), the median AUROC remained exceptionally stable across all thresholds: Type I ranged from 0.95 to 0.97 (IQR 0.02–0.03), Type I.5 from 0.94 to 0.96 (IQR 0.02–0.04), and Type II from 0.93 to 0.95 (IQR 0.03–0.04). The corresponding AUPR and MCC values showed similarly narrow ranges (e.g., Type I AUPR 0.74–0.79, MCC 0.63–0.69). This stability demonstrates that the strong evolutionary and coevolutionary signals anchoring the catalytic cleft are robustly captured regardless of whether the boundary includes only core coordinating residues or an expanded spatial shell.

In contrast, distal Type IV allosteric sites consistently exhibited a depressed and diffuse predictive profile across all thresholds. At the strictest cutoff (3.5 Å), median AUROC was 0.66 (IQR 0.13), median AUPR 0.16 (IQR 0.12), and median MCC 0.11 (IQR 0.14). When the pocket definition was expanded to a permissive 5.0 Å, these metrics shifted only marginally: AUROC to 0.68 (IQR 0.15), AUPR to 0.20 (IQR 0.14), and MCC to 0.12 (IQR 0.15). Proximal Type III allosteric sites also showed little variation, with AUROC ranging from 0.89 to 0.91 and MCC from 0.35 to 0.43 across thresholds (Table S6). This flat performance trajectory confirms that the PLM limitations to resolve allosteric binding sites with high confidence are independent of boundary placement, directly supporting our hypothesis that the allosteric blind spot arises from a diffuse embedding within the neutral‐frustration landscape rather than from an arbitrary choice of coordinate cutoffs.

To decouple loop flexibility from intrinsic pocket detectability, we recalculated all evaluation metrics after completely masking out the activation loop and αC‐helix segments across all human kinase structures (Table S7). Table S7 records model evaluation metrics at the baseline contact distance threshold under two conditions: (1) full‐length sequence evaluation, and (2) loop‐excluded evaluation, where all positions belonging to the activation loop and the C‐helix are fully omitted from metric calculations to isolate rigid versus flexible structural components. For orthosteric sites, median AUROC and MCC remained virtually unchanged after loop exclusion (e.g., Type I: AUROC 0.97 vs. 0.97, MCC 0.69 vs. 0.70; Type I.5: 0.96 vs. 0.95, 0.64 vs. 0.63; Type II: 0.95 vs. 0.95, 0.58 vs. 0.59). The results of this segment‐exclusion analysis reveal that the performance distributions are highly robust to loop omission. For proximal Type III allosteric sites, median AUROC was 0.91 both before and after loop exclusion (MCC 0.41 vs. 0.39). Hence, for Type III allosteric inhibitors which bind adjacent to the ATP cleft and directly interact with the C‐helix, the exclusion of these dynamic segments resulted in a minor contraction of the interquartile range (baseline AUROC vs. loop‐excluded), while the median AUROC remained essentially unchanged (baseline 0.81 vs. loop‐excluded 0.82) (Table S7). For distal Type IV allosteric sites, median AUROC remained 0.68 and MCC 0.12 versus 0.11, showing no statistically significant variation (*p* > 0.3, Mann–Whitney *U* test; Table S7). Collectively, these rigorous sensitivity evaluations demonstrate that residue‐level mapping choices do not distort or introduce artifactual bias into the benchmarking results. The allosteric blind spot is an invariant, reproducible, and robust feature of sequence‐based kinase architecture. Collectively, these rigorous sensitivity evaluations demonstrate that residue‐level mapping choices do not distort or introduce artifactual bias into the benchmarking results. The allosteric blind spot remains a reproducible outcome of sequence‐based PLM predictions on human kinases which is invariant to alternative definitions of pocket boundaries and masking of flexible loop regions.

### 2.5 Deconvolving label heterogeneity: granular pocket evaluation preserves detectability patterns and suggests an invariant signature of the allosteric blind spot

A critical question in AI benchmarking of functional sites is whether broad evaluation labels mask structural subpockets, thereby introducing label heterogeneity bias. In protein kinases, orthosteric inhibitors (Types I, I.5, and II) bind within a single, highly uniform spatial region: the canonical ATP‐binding cleft. By contrast, allosteric regulators (Types III and IV) target several distinct, non‐overlapping surfaces distributed across both the N‐ and C‐lobes of the catalytic core. Evaluating such multi‐modal spatial distributions under a single broad label could mechanically depress performance scores, potentially creating an artificial blind spot independent of any physical reality. To resolve this, we decomposed the broad allosteric classes into highly granular, spatially homogeneous subpockets that contain sufficient sample sizes within our dataset and reevaluated the fine‐tuned PLM predictions on these isolated subsets (Table S8). If the lower performance of allosteric sites were an artifact of label pooling, isolating a single uniform pocket, such as the myristoyl pocket in ABL kinase, would result in a sharp performance recovery, narrowing the gap with the orthosteric baseline.

The results of this subpocket stratification demonstrate that limited detectability may be an intrinsic property of each individual allosteric site. For proximal Type III allosteric sites, when broken down into the C‐Helix adjacent/DFG‐back pocket (Subtype III‐A) the PLM yielded predictions with a median AUROC of 0.90 and AUPR of 0.34 which are essentially indistinguishable from the pooled Type III baseline (AUROC 0.91, AUPR 0.36). As expected, the PLM predictions revealed reduced precision for the C‐Lobe distal myristoyl pocket (Subtype IV‐A), which is targeted by known ABL kinase allosteric drugs (Figure 2) with a median AUROC of 0.67 (IQR 0.11) and a median AUPR of 0.10 (IQR 0.05). These scores are statistically similar to the global Type IV category averages (median AUROC 0.68, median AUPR 0.08; Mann–Whitney *U* test, *p* > 0.3). Even when focusing exclusively on the ABL1/ABL2 myristoyl subset (*n* = 14), the performance remained similarly depressed (AUROC 0.65, AUPR 0.16). For the N‐Lobe hydrophobic PDK1‐interacting fragment (PIF)‐pocket (Subtype IV‐B), the PLM exhibited an equally flat profile, with a median AUROC of 0.69 (IQR 0.14) and a median AUPR of 0.07 (IQR 0.03) (Table S8). The observed persistence of limited detectability across highly granular allosteric subpockets indicates that the performance drop at allosteric sites is unlikely an artifact of label definitions or statistical pooling. Whether an allosteric pocket is located on the outer edge of the N‐lobe (PIF pocket) or buried at the base of the C‐lobe (myristoyl pocket), its residue embeddings consistently lack the concentrated, highly funneled sequence coevolution signatures that characterize the ATP cleft. In traditional classification tasks, resolving label heterogeneity by breaking down broad categories into uniform sub‐classes typically yields a noticeable boost in precision and recall. The fact that the model metrics remain flat even when focusing exclusively on highly uniform sites points to a deeper biological cause: the sequence embeddings themselves may carry fundamentally different signals for catalytic and allosteric spaces.

This observation naturally leads to the question: what physical property of these diverse allosteric subpockets explains their uniformly low detectability? In the following section, we turn to local frustration analysis—an independent, physics‐based energy landscape metric—to show that each of these individual subpockets is embedded within a region of neutral frustration. According to our conjecture, this energetic neutrality could preserve structural plasticity and regulatory versatility but may systematically erode the sequence‐based signals that PLMs rely upon, thereby providing a plausible biophysical explanation for the allosteric blind spot.

### 2.6 Local frustration analysis reveals landscape‐encoded determinants of prediction dichotomy between orthosteric and allosteric binding sites

The distribution of five classification metrics (AUROC, AUPR, MCC, Accuracy, and F1 score) for PLM predictions revealed reproducible signatures that differentiate site classes. The tight, tall distributions of orthosteric sites indicate that these sites are universally detectable as their signal is strong and invariant across structures. The broader, more variable distributions of Type III sites suggest that detectability depends on structural context. The extremely broad, collapsed distributions of Type IV sites suggest that these sites are only weakly detectable in most structures. These observations raise a fundamental question: What physical property of the sites themselves could explain these systematic differences in algorithmic detectability? We propose that the limitations of the PLM in detecting allosteric binding sites arise from a fundamental consequence of the intrinsic biophysical design of these sites.

To provide a mechanistic and physically grounded rationale for the divergent performance of the PLM on orthosteric versus allosteric kinase binding sites, we introduce local frustration analysis as a biophysical interpretability layer that connects algorithmic behavior to underlying physical properties of protein structures. Frustration analysis partitions residue–residue interactions into three energetic classes: minimally frustrated, neutrally frustrated, and highly frustrated based on how the native interaction energy compares to an ensemble of decoys generated either by randomizing residue identities (mutational frustration) or by perturbing both identities and local geometry (conformational frustration) (Chen et al., 2020; Ferreiro et al., 2007; Freiberger et al., 2021; Gianni et al., 2021; Parra et al., 2016, 2025). For minimally frustrated contacts, native interactions are strongly favored over alternatives; neutrally frustrated contacts are characterized by native interactions that are neither strongly favored nor disfavored; and for highly frustrated contacts, native interactions are less favorable than alternative decoys pointing to significant local strain (Ferreiro et al., 2007; Parra et al., 2016).

**FIGURE 4:**
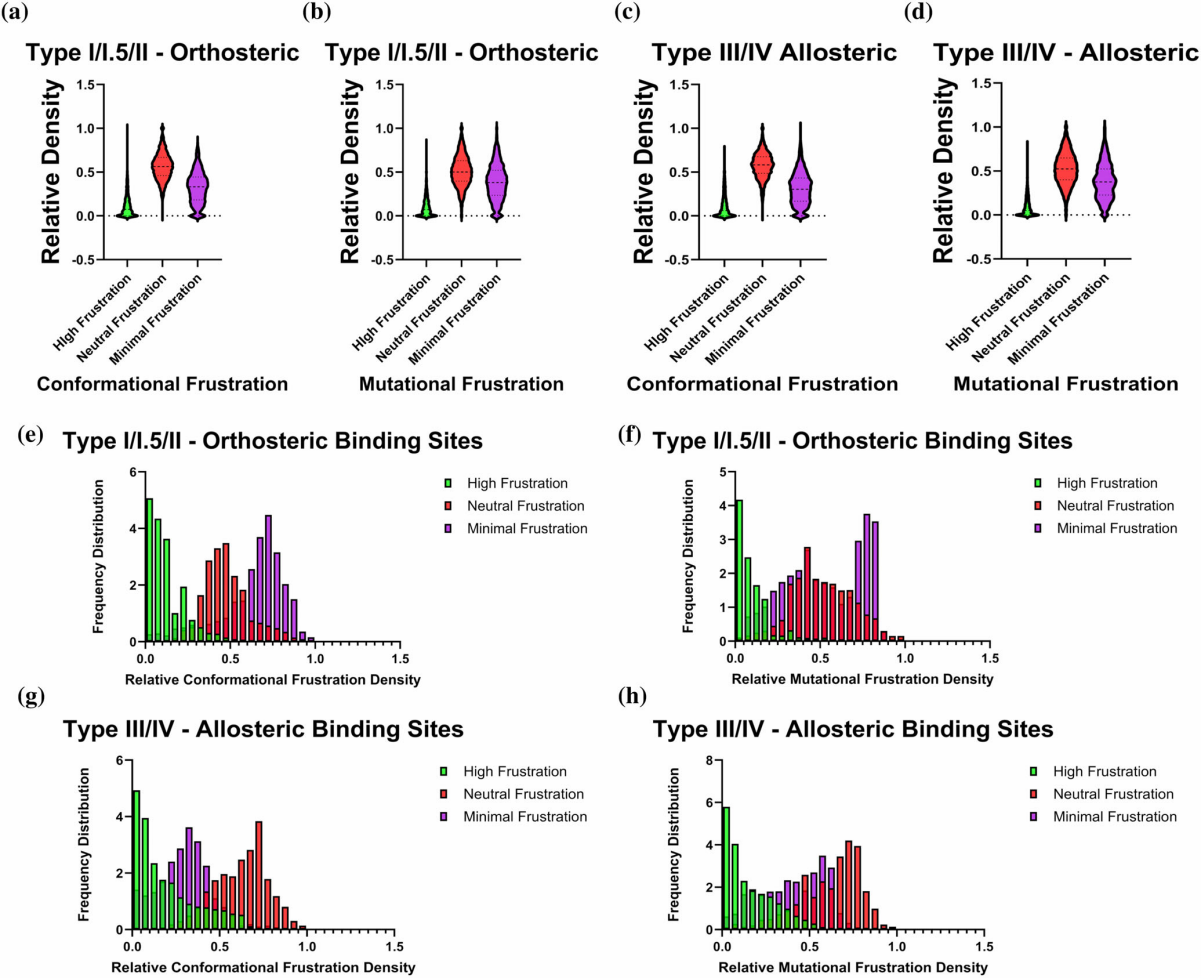
Distinct frustration landscapes encode the predictability of orthosteric versus allosteric kinase binding sites. (a–d) Global frustration architecture of the kinase domain. Violin plots show the relative density of frustration classes for the entire kinase domain fold, independent of binding site location. Panels display conformational frustration (a, c) and mutational frustration (b, d) for complexes with orthosteric inhibitors (a, b) and allosteric modulators (c, d). The distributions are nearly superimposable across both classes, dominated by neutral frustration (red), indicating that the global energetic scaffold of the kinase is conserved and intrinsically plastic regardless of ligand type. (e–h) Binding site‐specific frustration distributions are show in color‐coded filled bars: Green for high frustration (energetically strained); red for neutral frustration (energetically indifferent); and purple for minimal frustration (energetically optimized). Histograms display the frequency distribution of relative frustration density for residues strictly within annotated ligand‐binding pockets. Frustration profiles for orthosteric binding sites (e, f) exhibit a pronounced skew toward minimal frustration (purple), particularly for mutational frustration (f), where the distribution peaks at high densities (>0.7). In stark contrast, allosteric binding sites (g, h) are overwhelmingly dominated by neutral frustration (red) with a minor but consistent population of high frustration (green). The near‐absence of minimal frustration in allosteric pockets indicates evolutionary permissiveness and conformational adaptability, explaining their statistical invisibility to sequence‐based models.

Here we assess the hypothesis that predictive success is governed by the underlying frustration signature of a functional site. We began by examining the global frustration landscape of the KD. For each structure in our dataset, we computed both conformational frustration (sensitivity to structural perturbations) and mutational frustration (sensitivity to amino acid substitutions) and visualized the distributions across all residues (Figure 4a–d). Strikingly, the global frustration profiles of orthosteric and allosteric kinase complexes were nearly indistinguishable in both conformational frustration (Figure 4a,c) and mutational frustration regimes (Figure 4b,d). Conformational frustration distributions show a dominant, broad peak centered in the neutral‐frustration regime, indicating that most interactions throughout the kinase fold are neither strongly optimized nor strongly strained. The violin plots are wide and roughly symmetric, reflecting the heterogeneous mix of structural elements that comprise the KD (Figure 4a,c). A similar pattern emerges for mutational frustration where the majority of residues also fall within the neutral regime, with the distributions tapering off gradually toward both minimal and highly frustrated extremes (Figure 4b,d). This similarity reveals that the global energetic architecture of the kinase catalytic domain is largely conserved. The KD fold provides a shared platform where neutral frustration predominates, enabling the conformational flexibility required for transitions between active and inactive states.

When we restrict analysis to residues within annotated binding pockets, a strikingly different picture emerges, and here the shapes of the distributions become powerfully diagnostic. Orthosteric binding sites exhibit distributions that are sharply shifted toward minimal frustration, with a pronounced peak at high minimal frustration densities (>0.7) (Figure 4e,f). The histogram peaks sharply in the minimal frustration regime, with relative densities reaching their maximum at high minimal frustration values (>0.7) and tapering off rapidly in the neutral and highly frustrated regimes (Figure 4e). This is the signature of intense purifying selection: evolution has locked these residues in place because catalytic function demands precise geometry. The enrichment is even more dramatic for mutational frustration (Figure 4f), where the distribution is almost entirely confined to the minimal regime, reflecting the strong destabilization that would result from amino acid substitutions at these positions.

Conformational frustration regime for allosteric binding sites (Figure 4g) exhibits a fundamentally different histogram shape. Here the distribution peaks squarely in the neutral‐frustration regime (0.65–0.75), with minimal frustration densities markedly reduced compared to orthosteric sites. The histogram is broader and more evenly distributed, indicating that allosteric sites are evolutionarily permissive; many substitutions are energetically tolerated, enabling sequence drift and conformational plasticity without compromising function. Mutational frustration distributions for allosteric site residues (Figure 4h) reinforce and amplify this dichotomy. While for orthosteric sites the mutational frustration histogram is almost entirely confined to the minimal regime, the distribution for allosteric sites shifts dramatically toward neutral frustration, with minimal frustration nearly absent. Notably, both conformational and mutational frustration histograms for allosteric sites show small but consistent tails extending into the highly frustrated regime.

To test whether this frustration signature is consistent across individual allosteric subpockets and to directly link frustration states to PLM classification outcomes, we analyzed the ABL kinase structures in detail, mapping the model residue‐level prediction outcomes across all ABL structures directly to independent physical frustration states, showing how the model sequence‐level predictions correspond to distinct physical environments (Table S9).

For True Positives (TP) predictions, that is, residues correctly identified as part of the orthosteric ATP cleft, the majority (68.5%) are minimally frustrated, with only 22.1% neutral and 9.4% highly frustrated. In contrast, False Negatives (FN) which are residues belonging to the distal allosteric myristoyl pocket that the PLM failed to detect show the opposite pattern: only 8.4% minimally frustrated, 78.9% neutrally frustrated, and 12.7% highly frustrated (Table S9). This holds for both broad myristoyl pockets and the ABL1/ABL2‐specific subset. TN (the rigid structural core) are overwhelmingly minimally frustrated (82.3%), while False Positives (FP) (flexible surface loops) exhibit a mixed profile (12.5% minimal, 46.0% neutral, and 41.5% highly frustrated), consistent with their conformational mobility (Table S9).

Critically, the granular subpockets Subtype IV‐A and Subtype IV‐B individually display a similar neutral frustration dominance as the entire pooled Type IV category. This uniformity across distinct allosteric subpockets further reinforces the assertion that the reduced detectability even for homogeneous subsets of allosteric pockets may be associated with an intrinsic biophysical organization of energy landscapes for allosteric regions rather than present an artifact of label pooling or structural heterogeneity.

The contrasting shapes of the frustration histograms for orthosteric and allosteric binding site residues suggest a plausible physical rationale for the observed PLM performance patterns. The sharp peaks of orthosteric sites may be associated with deep, funneled energetic basins that generate strong, unambiguous coevolutionary signals PLMs detect with high confidence. In some contrast, the broad, neutral‐dominated histograms of allosteric sites could reflect shallow, rugged landscapes where evolutionary signals are likely weak and variable, which is consistent with the diffuse, low‐confidence and structure‐dependent detectability observed for Type III and IV sites.

### 2.7 Architectural patterns of neutral frustration across the kinome

The quantitative dichotomy raises two interrelated questions about spatial organization. Where are allosteric sites located across the kinase fold and where does neutral frustration localize within kinase structures? To address these questions, we performed complementary mapping analyses that together reveal a striking convergence. To visualize the structural distribution of neutral frustration, we mapped high‐density neutrally frustrated positions onto the KDs of multiple representative kinases bound to various allosteric ligands (Figure S4). Each panel displays a different kinase–ligand complex, with neutrally frustrated residues rendered in blue. Despite the diversity of kinases and ligands, a consistent architectural pattern emerges that directly parallels the allosteric site map (Figure S4). The ATP‐binding cleft, the canonical activation segment, and the central hydrophobic spine residues essential for catalysis exhibit minimal frustration density, consistent with their evolutionary constraint: these elements must maintain defined conformations to support phosphotransfer activity and are therefore under strong purifying selection. Neutral frustration consistently localizes to peripheral and conformationally adaptable regulatory regions—flexible loops, dynamic hinges, and lineage‐specific pockets—while being systematically excluded from the conserved catalytic core (Figure S4). This extended mapping of high neutral frustration density on representative kinase complexes with Types III and IV allosteric inhibitors reinforces the notion that regulatory potential may be encoded in regions of the energy landscape that are evolutionarily permissive and conformationally plastic.

Across all complexes, neutral frustration consistently decorates three classes of regions: flexible regulatory loops (activation loop, P + 1 motif), dynamic mechanical hinges (αC‐helix, DFG motif, lobe connector), and lineage‐specific allosteric pockets (myristoyl site, DFG‐out pocket, and C‐lobe regulatory interfaces) (Figure S4). This corresponds to the location of several characterized allosteric binding interfaces, and the neutral frustration of the P + 1 motif likely reflects its need to accommodate diverse substrate sequences while maintaining recognition specificity. Similarly, neutral frustration consistently decorates the hinges that connect rigid elements, such as the αC‐helix, which swings between “in” and “out” conformations to control catalysis, and the DFG motif, which flips to gate ATP and inhibitor binding (Figure S4). The key finding of this analysis is that the neutral frustration density overlaps significantly with both proximal (Type III) and distal (Type IV) allosteric sites. In ABL, the myristoyl pocket in the C‐lobe is clearly delineated by high neutral frustration density (Figure S4). A significant neutral frustration in the allosteric pockets reflects this evolutionary history: they remain plastic, enabling each kinase family to develop specialized regulatory interfaces while preserving the underlying conformational flexibility required for function. The neutral frustration density is not necessarily connected as a single continuous surface. In many structures, it appears as multiple distinct small clusters distributed across the KD—a cluster in the A‐loop, another around the αC‐helix hinge, a third in the myristoyl pocket. The minimally frustrated catalytic core must remain rigid to maintain catalytic geometry, while neutral frustration is strategically deployed at regulatory junctions where conformational transitions occur. This creates an energetic mosaic where stable cores are interspersed with flexible switches. This discontinuous organization suggests that neutral frustration is a local feature of specific structural elements that may have evolved for conformational flexibility. However, these spatially separated neutral frustration clusters do not function in isolation; rather, they form interconnected nodes within allosteric communication networks that couple distal regulatory sites to the catalytic center.

Drawing from our previous studies of allosteric interaction networks in protein kinase structures and complexes (Dixit & Verkhivker, 2011a, 2011b; Krishnan et al., 2022; Raisinghani et al., 2024; Tse & Verkhivker, 2015a, 2015b) as well as other theoretical studies (Nussinov et al., 2025) and a significant body of nuclear magnetic resonance experiments (Kim et al., 2017; Masterson et al., 2012; Olivieri et al., 2021) the discontinuous but organized distribution of frustration clusters can reflect their integration into allosteric communication networks: stable positions provide reliable signal transmission pathways, while neutrally frustrated residues enable the conformational flexibility required for network switching. This hybrid architecture in which rigid backbone elements interspersed with frustrated switching points can transform local ligand binding into global regulatory outcomes, allowing the kinase to integrate allosteric signals into a functional response.

While neutral frustration dominates the stable kinase conformations, our previous studies also revealed a dynamic dimension to this architectural principle. We previously found that localized highly frustrated clusters in inactive kinase states could collocate with the regions directly involved in conformational changes associated with kinase function (Dixit & Verkhivker, 2011a, 2011b). This suggests a dual role for frustration in these regulatory regions. Neutral frustration establishes the baseline thermodynamic landscape that permits conformational sampling within stable functional states: this is the energetic signature of the “permissive scaffold” we observe across the KD. However, localized clusters of high frustration within these neutrally frustrated regions serve as conformational nucleation points that become particularly pronounced in higher‐energy inactive states, creating the strain necessary to drive large‐scale structural transitions. Taken together, these findings suggest that allosteric sites may be encoded in neutrally frustrated regions optimized for context‐dependent regulatory modulation while the capacity to accumulate high frustration in transition states provides an energetic driving force for conformational switching.

### 2.8 ABL kinase as a canonical system for decoding frustration signatures

The previous sections established two complementary findings at the kinome‐wide level. First, orthosteric and allosteric sites exhibit systematically different patterns of PLM detectability (Figure 3). Second, these differences in algorithmic behavior reflect underlying differences in energetic embedding: orthosteric sites are enriched in minimal frustration, while allosteric sites are dominated by neutral frustration and localize to peripheral regions of the kinase fold (Figure 4).

To anchor the frustration trends in a physically interpretable molecular system, we now examine these principles at atomic resolution using the ABL kinase system where the relationship between neutral frustration, conformational plasticity, and regulatory versatility can be explored in molecular detail across multiple ligand‐bound states and regulatory contexts. The regulatory myristoyl pocket in ABL (Figure 2) exemplifies the functional versatility of allosteric sites: the clinically approved drug Asciminib binds here to stabilize an autoinhibited conformation (Schoepfer et al., 2018; Wylie et al., 2017); Genomics Institute of the Novartis Research Foundation compound 2 (GNF‐2) and GNF‐5 also target this pocket but function as inhibitors (Zhang et al., 2010); the small molecule DPH acts as an activator (Yang et al., 2011); and the ATP‐competitive inhibitor Imatinib itself can bind to this pocket, where the interaction both competes with catalytic inhibition and promotes conformations associated with increased kinase activity (Xie et al., 2022). The wealth of experimental characterization enables us to test whether insights from kinome‐wide analysis hold up under detailed atomic scrutiny.

**FIGURE 5:**
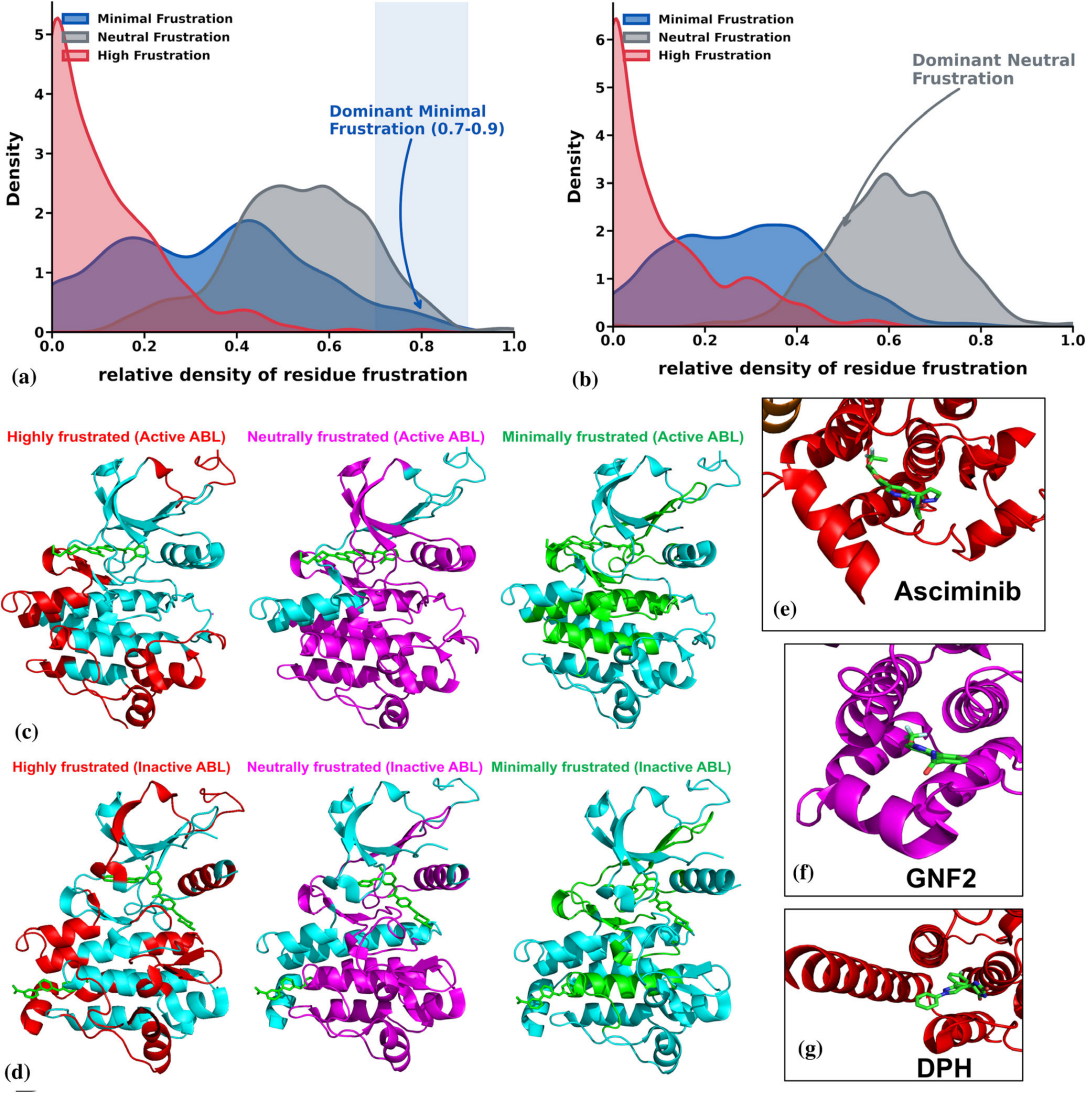
Atomic‐resolution frustration signatures of orthosteric and allosteric sites in ABL kinase reveal distinct energetic and structural fingerprints. (a) Cumulative probability density of conformational frustration for orthosteric binding site residues aggregated over all ABL complexes with orthosteric inhibitors. A pronounced high‐density region between 0.7 and 0.9 on the *x*‐axis (blue‐shaded background) is dominated by minimally frustrated interactions (deep blue curve), indicating strong energetic optimization of the ATP‐binding cleft. (b) Cumulative probability density of conformational frustration for allosteric binding site residues aggregated over all ABL complexes with allosteric inhibitors. The distribution is dominated by neutrally frustrated interactions (gray curve) and exhibits a shift toward higher frustration values, reflecting energetic permissiveness and conformational heterogeneity. (c) Structural mapping of local frustration hotspots in the ABL complexes bound to orthosteric Type I/II inhibitors (ligand shown in sticks; kinase domain in light pink ribbons). Highly frustrated hotspots (top 10%, red ribbons) localize predominantly to flexible regulatory regions, including the activation loop. Neutrally frustrated hotspots (top 10%, magenta ribbons) form a percolating scaffold throughout the fold, supporting conformational adaptability. Minimally frustrated hotspots (top 10%, green ribbons) cluster in the conserved ATP‐binding cleft and catalytic core, coinciding directly with the orthosteric binding site. (d) Structural mapping of frustration hotspots in inactive ABL complexes bound to the Type II orthosteric inhibitor and ABL allosteric inhibitors (both ligands shown in sticks). Highly frustrated hotspots (red ribbons) concentrate in regulatory switch regions. Neutrally frustrated hotspots (magenta ribbons) permeate the kinase domain and are enriched around the myristoyl allosteric pocket. Minimally frustrated hotspots (green ribbons) localize in the catalytic core and orthosteric site. (e) Close‐up view of the myristoyl allosteric pocket bound by the allosteric inhibitor Asciminib (ligand in atom‐colored sticks) showing pronounced bending of the αI‐helix associated with stabilization of the ABL autoinhibited conformation. (f) Close‐up view of the allosteric pocket bound by the inhibitor GNF‐2, highlighting a distinct ligand‐induced deformation that stabilizes an inactive conformation. (g) Close‐up view of the myristoyl pocket bound by the allosteric activator DPH, showing partial unbending of the αI‐helix that disrupts autoinhibitory constraints and promotes active ABL conformation.

Residue‐level frustration profiling was performed across an ensemble of ABL complexes spanning active, intermediate, and fully autoinhibited states bound to orthosteric and allosteric ligands, providing a structural‐scale test of the hypothesis that orthosteric and allosteric sites are encoded in fundamentally different energetic regimes (Figure 5). In ABL complexes bound to Type I ATP‐competitive inhibitors, the orthosteric binding cleft is strongly enriched in minimally frustrated residues, both conformationally and mutationally (Figure 5a). The probability density is sharply shifted toward the minimal‐frustration regime, with a pronounced population at high minimal‐frustration fractions (0.7–0.9), while maintaining a modest neutral‐frustration background.

This pattern defines an energetically locked interaction hotspot in which native residue identities and geometries are highly optimized and substitutions are strongly penalized. This architecture reflects strong evolutionary constraint and geometric recurrence, producing the conserved sequence and structural signatures that PLMs and structure‐based predictors recognize with high precision. In contrast, allosteric sites in ABL in complexes with allosteric modulators exhibit a fundamentally different frustration signature (Figure 5b). The probability density shifts toward neutral frustration, with a broader distribution and an increased contribution from highly frustrated residues. The results reflect the spatial distribution of allosteric pockets embedded within a scaffold of predominantly neutrally frustrated residues, punctuated by localized clusters of high frustration at regulatory junctions involving the αI‐helix, activation loop, and regulatory spine (Figure 5b).

Structural mapping of frustration hotspots in the ABL orthosteric complexes reinforces this interpretation (Figure 5c). Minimally frustrated residues concentrate in the catalytic core, including the central β‐sheet, DFG motif, histidine‐arginine‐aspartate motif (HRD) catalytic loop, and regulatory spine, defining a stability nucleus that enforces a conserved geometry for ATP binding and catalysis. Neutrally frustrated residues form a continuous scaffold linking the N‐ and C‐lobes, providing flexibility for hinge motion and αC‐helix repositioning. Highly frustrated residues cluster at known conformational switch regions, including the activation loop and αC‐helix interface, where strain is locally stored to facilitate state transitions. Thus, orthosteric recognition is embedded in a landscape that is globally plastic but locally locked at the binding site. Structural projection of frustration classes in the ABL allosteric complexes (Figure 5d) shows that highly frustrated residues form a contiguous frustrated region involving the A‐loop (residues 384–410), which folds inward to make extensive contacts with the αG‐helix (residues 425–445), and the intervening P + 1 motif (residues 404–412). This tripartite assembly is characterized by non‐native residue–residue contacts and energetic strain, and it plays a critical role in allosteric control by locking ABL in the inactive state. These highly frustrated clusters correspond to regions that undergo large structural rearrangements during inactive‐to‐active transitions, effectively serving as “initiation cracking points” that can perturb the inactive state and promote functional switching (Miyashita et al., 2003; Whitford et al., 2007). This analysis reinforces the notion of a dynamic frustration model in which dynamic regulatory regions that exhibit neutral frustration in dormant, low‐energy states could accumulate high frustration in more strained inactive conformations. We argue that this conformational dependence of frustration signatures has important implications for understanding allosteric site detectability. Allosteric pockets reside in regions that sample multiple frustration regimes across the conformational ensemble. In some states, these regions display neutral frustration (permitting conformational sampling); in others, they accumulate high frustration (priming transition). This frustration heterogeneity across states further erodes the consistent evolutionary signals that PLMs require for reliable detection. By contrast, orthosteric sites maintain minimal frustration across all conformational states, reflecting their role as stable anchoring points that must preserve catalytic geometry regardless of regulatory state. This frustration stability generates the robust coevolutionary signals that enable high‐precision detection.

To provide a more granular, atomic‐resolution illustration of the general frustration signatures described for orthosteric and allosteric binding sites, we present a detailed residue‐level analysis of high, neutral, and minimally frustrated positions across distinct ABL kinase complexes: (a) the active and inactive catalytic domain conformations formed by Type I kinase inhibitors Dasatinib (Protein Data Bank [PDB] id 2GQG) (Tokarski et al., 2006) and Axitinib (PDB id 4WA9) (Pemovska et al., 2015); (b) the inactive ABL structure bound with Type II inhibitor Imatinib and allosteric inhibitor GNF‐2 (PDB id 3K5V) (Zhang et al., 2010), inactive ABL complex with Type II Imatinib and allosteric activator DPH (PDB id 3PYY) (Yang et al., 2011) and the autoinhibitory ABL regulatory complex with Type II inhibitor Nilotinib and allosteric inhibitor Asciminib (PDB id 5MO4) (Panjarian et al., 2013; Schoepfer et al., 2018). The residue‐based frustration profiling of ABL kinase complexes with the Type I inhibitors Dasatinib (Figure S5) and Axitinib (Figure S6) provides a specific, atomic‐resolution illustration of the general frustration signatures described for orthosteric binding sites. Conformational frustration and mutational frustration profiles for these complexes display the canonical energetic signature of an optimized orthosteric pocket. In the Dasatinib complex, the ATP‐binding cleft (residues 315–321) is dominated by minimally frustrated residues, forming a stable, low‐frustration interaction core. Concurrently, discrete clusters of high frustration are localized in regulatory regions, including the C‐lobe (residues 320–340), the A‐loop and P + 1 loop (residues 395–410), and the αG‐helix (residues 440–455). These high‐frustration zones correspond to conformationally adaptable motifs that are implicated in functional switching (Figure S5). A nearly identical frustration pattern is observed in the Axitinib complex, where the orthosteric site is similarly enriched in minimal frustration, consistent with its conserved hydrogen‐bonding network (e.g., with T315, K271, Y253, and F382) (Figure S6). Notably, the mutational frustration profiles for both inhibitors show an even stronger concentration of minimal frustration in the binding site than the conformational profiles, highlighting the evolutionary conservation and sequence constraint characteristic of orthosteric pockets. These results directly exemplify the “energetically locked” architecture described for Type I inhibitors in the main text: the orthosteric site is a hotspot of minimal frustration reflecting evolutionary optimization and structural invariance, whereas surrounding regulatory elements exhibit elevated frustration, enabling the conformational plasticity required for allosteric regulation and state transitions.

### 2.9 Dynamic frustration signatures of the ABL inactive states: persistent neutrality of myristoyl pocket and shift to high frustration in regulatory sites mediating transitions

Allosteric regulation introduces a distinct energetic signature characterized by persistent neutral frustration. Even in the maximally stabilized, autoinhibited assembly bound to both Nilotinib and Asciminib (Figure S7), the allosteric myristoyl pocket remains heavily neutrally frustrated, suggesting this region is evolutionarily “concealed” to maintain functional plasticity. This persistent neutral frustration signature is similarly observed in the KD when bound to the foundational allosteric inhibitor GNF‐2 (Figure S8). Notably, this neutral landscape is not restricted to inhibited states; the allosteric activator DPH (Figure S9) also targets a region of high neutral frustration density, further confirming that these regulatory pockets lack the strong coevolutionary optimization seen at orthosteric sites. Importantly, these complexes consistently display a predominance of neutrally frustrated residues enriched in and around the allosteric myristoyl pocket. This pattern contrasts sharply with the minimal‐frustration signature of orthosteric sites and highlights the evolutionary plasticity and structural adaptability of allosteric regions. Despite the overall prevalence of neutral frustration, we observed appreciable redistribution from neutrally frustrated to highly frustrated clusters in key regulatory junctions implicated in functional kinase transitions (Figures S7–S9). Overall, the frustration signatures of allosteric ABL complexes illustrate an adaptive energetic regime: a backbone of neutral frustration that permits conformational flexibility, interspersed with accumulation of high‐frustration clusters at regulatory interfaces in inactive states, creating a driving force for conformational switching.

The important finding of this analysis is the seemingly persistent neutrality of the allosteric myristoyl pocket across different ABL states and complexes. Although Asciminib, GNF‐2, and an allosteric activator 3‐(4‐fluorophenyl)‐1‐phenyl‐1H‐pyrazole‐4‐carbaldehyde (DPH) occupy the same myristoyl pocket, each induces a distinct local conformational response, demonstrating that the pocket is not a fixed cavity but a deformable regulatory interface whose geometry is reshaped by ligand‐dependent redistribution of frustration. Upon Asciminib binding, the αI‐helix undergoes a pronounced bending motion that stabilizes the autoinhibited state (Figure 5e). The residues accommodating this deformation are largely neutrally frustrated, with embedded highly frustrated positions acting as mechanical pivots that enable strain redistribution without enforcing strict sequence conservation. Binding of GNF‐2 produces a different deformation of the same helix and surrounding regulatory elements, yielding an alternative inactive configuration (Figure 5f). Compared with Asciminib, the magnitude and direction of helix bending differ, and adjacent loops and spine elements reorganize in a ligand‐specific manner. In contrast, binding of the allosteric activator DPH partially reverses this deformation, unbending the αI‐helix and promoting a more active‐like configuration (Figure 5g). This inversion of response‐activation rather than inhibition demonstrates that the same pocket can support opposing functional outcomes. These differences show that the pocket does not encode a single canonical inhibitory geometry but supports multiple low‐energy conformations depending on how local frustration is relieved.

Combined, these structural responses of the ABL allosteric sites to binding of different modulators exemplify the defining feature of the allosteric sites across the human kinome characterized by context‐dependent remodeling. Neutral frustration of the energy landscapes for the allosteric binding regions ensures mutational tolerance and conformational diversity, while localized high frustration enables sensitivity to perturbation and signal propagation. The ABL analysis provides atomic‐scale validation of the global frustration–predictability relationship. Orthosteric sites are encoded as energetically optimized basins, producing conserved sequence and geometric patterns that PLMs and structure‐based methods detect with high confidence. By contrast, allosteric sites are encoded as energetically permissive and heterogeneous regions whose defining features are conformational strain and context‐dependent remodeling rather than conserved motifs. The invisibility of allosteric pockets to AI predictors is not accidental but emerges from their energetic architecture: neutral frustration masks evolutionary regularities while localized high frustration encodes regulatory sensitivity.

### 2.10 Conserved Island of neutrality: neutral frustration of ABL myristoyl allosteric pocket persists across distinct allosteric modulators and regulatory domains

**FIGURE 6:**
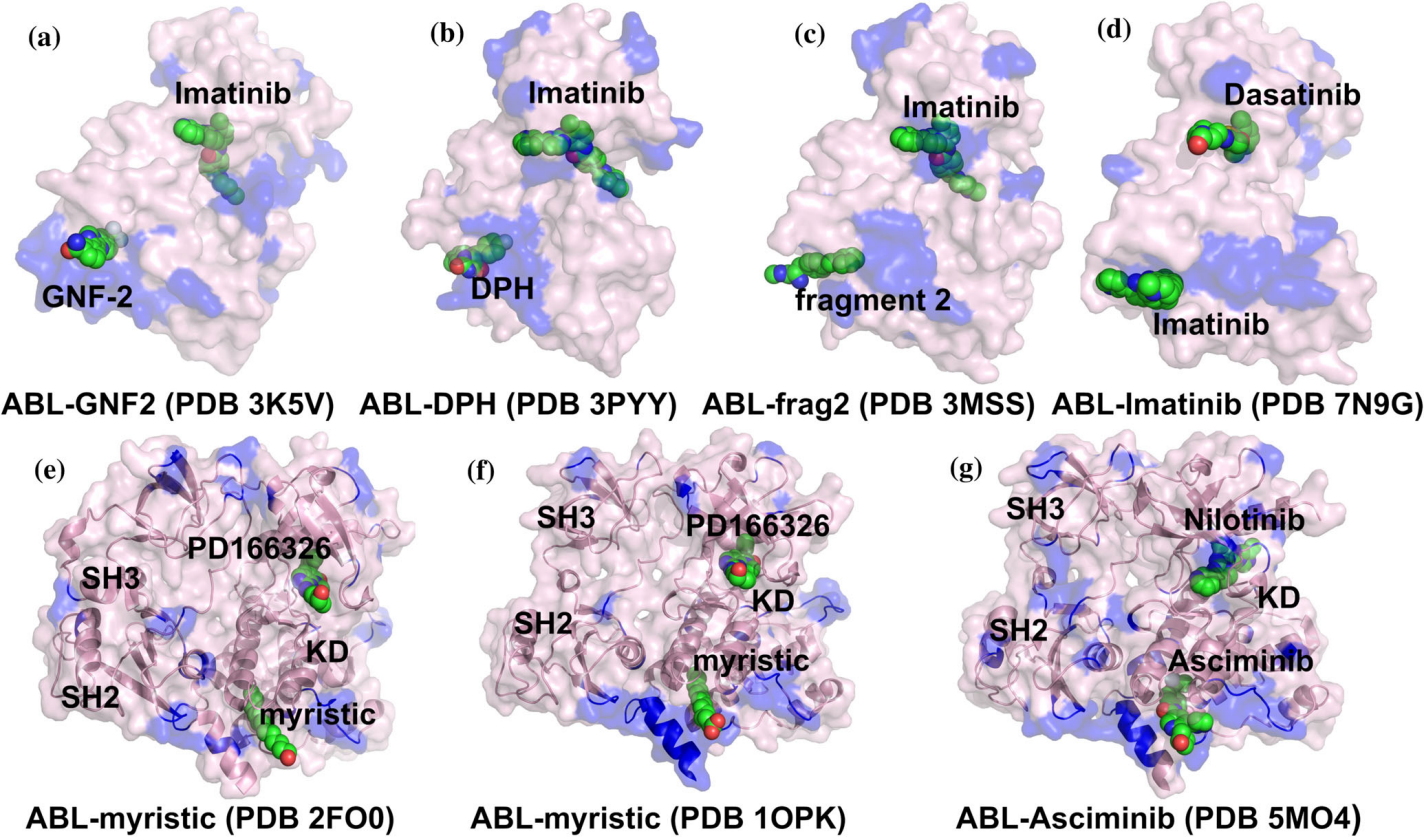
Structural diversity and persistent neutral frustration signatures at the ABL myristoyl allosteric pocket across regulatory states. (a–d) Isolated kinase domain complexes with diverse allosteric modulators. Surface representations of the ABL kinase domain bound to distinct ligands in the orthosteric and allosteric sites. Blue patches indicate regions of high‐density neutral frustration. (a) ABL bound to the allosteric inhibitor GNF‐2 (green spheres) in the myristoyl pocket and Imatinib (orthosteric) in the ATP cleft (Protein Data Bank [PDB] 3K5V). (b) ABL bound to the allosteric activator DPH (green/red spheres) in the myristoyl pocket and Imatinib (orthosteric) (PDB 3PYY). (c) ABL bound to allosteric fragment 2 (green spheres) and Imatinib (orthosteric) (PDB 3MSS). (d) ABL bound to Imatinib (green spheres) in the allosteric pocket and Dasatinib (orthosteric) in the ATP cleft, illustrating allosteric binding of an ATP‐competitive inhibitor in resistant variants (PDB 7N9G). In all cases, the allosteric pocket is lined by neutral frustration hotspots (blue), reflecting conformational plasticity. (e–g) Full SH3–SH2–kinase domain (KD) regulatory assemblies. Ribbon and surface representations of the autoinhibited ABL assembly, showing the SH3 (dark blue), SH2 (light blue), and Kinase Domain (pink/white) domains. Blue patches on the kinase domain surface indicate neutral frustration density. (e) Autoinhibited assembly with myristic acid (green/red spheres) bound in the allosteric pocket and PD166326 (orthosteric) (PDB 2FO0). (f) Autoinhibited assembly with myristic acid bound in the allosteric pocket and PD166326 (orthosteric) (PDB 1OPK). (g) Autoinhibited assembly with the allosteric inhibitor Asciminib (green/red spheres) bound in the myristoyl pocket and Nilotinib (orthosteric) (PDB 5MO4).

To investigate how neutral frustration behaves under complex regulatory conditions that more closely mirror the cellular context, we performed comparative frustration mapping on three distinct structural states of the ABL kinase that include the SH2–SH3 regulatory domains (Figure 6). This series captures the ABL kinase across key regulatory states from the unliganded autoinhibited assembly through the myristoyl‐stabilized inactive conformation to inhibitor‐bound forms, providing a window into how frustration signatures respond to both protein–protein interactions and small‐molecule binding. We first examined isolated ABL KD complexes bound to chemically and functionally diverse allosteric modulators (Figure 6a–d). Despite substantial differences in ligand chemistry and functional outcomes, the myristoyl pocket consistently displays high‐density neutral frustration (blue patches) across all complexes. In the ABL‐GNF‐2 complex (Figure 6a), the allosteric inhibitor GNF‐2 binds the myristoyl pocket while Imatinib occupies the orthosteric ATP cleft.

The pocket‐lining residues exhibit prominent neutral frustration density, reflecting the conformational plasticity that enables GNF‐2 to stabilize the autoinhibited state through αI‐helix bending. Similarly, in the ABL‐DPH complex (Figure 6b), the allosteric activator DPH binds the same myristoyl pocket yet produces the opposite functional outcome—promoting an active‐like conformation through αI‐helix unbending. Despite this functional inversion, the pocket retains its neutral frustration signature, indicating that the underlying energetic architecture permits both inhibitory and activating conformational responses. The ABL‐fragment 2 complex (Jahnke et al., 2010) (Figure 6c) demonstrates that even small molecular fragments promote an extended active‐like conformation and exert dual negative effects on inhibition; fragments binding the myristoyl pocket encounter the same neutrally frustrated environment. Most strikingly, in the ABL‐Imatinib complex (Figure 6d), Imatinib—originally designed as an ATP‐competitive inhibitor—binds the allosteric myristoyl pocket in drug‐resistant variants, while Dasatinib occupies the orthosteric site. This unexpected allosteric binding mode occurs within the same neutrally frustrated pocket architecture observed with dedicated allosteric modulators. The critical observation across all four complexes is that the allosteric pocket is consistently lined by neutral frustration hotspots (blue patches), regardless of ligand identity or functional outcome. This persistence indicates that neutral frustration is not a consequence of specific ligand chemistry but an intrinsic property of the pocket that enables its regulatory versatility and robust adaptation to different allosteric inhibitors and activators.

To determine whether neutral frustration persists in more physiologically relevant regulatory contexts, we examined full SH3–SH2–KD autoinhibited assemblies (Figure 6e–g). These complexes include the upstream regulatory domains that stabilize the autoinhibited state through interdomain contacts, providing a more complete picture of how the myristoyl pocket functions within the native regulatory architecture. In the apo SH3–SH2–KD assembly (Figure 6e), the myristoyl pocket is occupied by endogenous myristic acid while PD166326 binds the orthosteric site. The SH3 and SH2 domains clamp the KD in the autoinhibited conformation. Despite this extensive interdomain stabilization, the myristoyl pocket retains its characteristic neutral frustration density (blue patches), extending into the adjacent αI‐helix that mechanically couples the pocket to the SH2‐kinase interface. The same pattern holds in the SH3–SH2–KD assembly with myristic acid and PD166326 (Figure 6f), where the regulatory domains and orthosteric inhibitor jointly stabilize the inactive state. The myristoyl pocket remains neutrally frustrated, indicating that even when the pocket is occupied by its physiological ligand and the kinase is fully autoinhibited, the pocket retains conformational plasticity rather than settling into a single rigid minimum. In the SH3–SH2–KD–Asciminib–Nilotinib complex (Figure 6g), the clinically approved allosteric inhibitor Asciminib binds the myristoyl pocket while Nilotinib occupies the orthosteric site, with the SH3–SH2 clamp maintaining autoinhibition. Despite simultaneous engagement of both orthosteric and allosteric sites by clinical inhibitors, and despite the stabilizing influence of the SH3–SH2 regulatory domains, the myristoyl pocket and αI‐helix retain their neutral frustration signature.

Taken together, the examined seven complexes establish the ABL myristoyl pocket as a paradigmatic example of intrinsically encoded and persistent neutral frustration. Across isolated KDs and full regulatory assemblies, across diverse ligands (GNF‐2, DPH, fragment 2, Imatinib, myristic acid, Asciminib), and across functional outcomes (inhibition, activation, and resistance), the pocket maintains its neutral frustration signature. The allosteric pocket is integrated into broader allosteric networks that connect this distal site to the ATP‐binding cleft, ensuring that local binding events propagate across the entire structure. This architectural design enables the pocket to serve its biological function: remaining continuously available for context‐dependent modulation while providing a druggable target for allosteric inhibitors. The ABL analysis provides atomic‐resolution validation of the principles inferred from kinome‐wide data. The enrichment of minimal frustration at orthosteric sites, the dominance of neutral frustration at allosteric sites, the spatial segregation of frustrated regions from the catalytic core, and the persistence of neutral frustration across diverse regulatory states mirror and reinforce the patterns observed across the broader kinase family.

## 3 DISCUSSION

The results of this study indicated that binding site detectability by PLM correlates with the local frustration class of the site. Orthosteric sites are enriched in minimally frustrated residues and are detected with high precision, while allosteric sites are enriched in neutral frustration and are detected with markedly reduced confidence. This correlation is consistent with the hypothesis that energy landscape organization influences algorithmic visibility. By repurposing PLMs from predictive tools into instruments for interrogating evolutionary design, we show that these models may be intrinsically biased for convergence to minimally frustrated regions which are associated with structurally constrained, catalytically essential cores shaped by evolutionary selection. In contrast, allosteric sites consistently localize within neutrally frustrated environments that enable conformational plasticity and mutational tolerance. This evolutionary permissiveness could weaken sequence‐level constraints and erode the statistical signals required for inference, giving rise to an intrinsic allosteric blind spot. Protein kinases provide a uniquely well‐controlled system for uncovering this relationship. The extensive experimental foundation ensures that the observed dichotomy between orthosteric and allosteric detection arises from genuine energetic and evolutionary principles rather than dataset bias. The availability of diverse structural ensembles further enables frustration analysis to capture the energetic determinants of conformational transitions central to kinase regulation.

A key advance of this work is the introduction of local frustration analysis as an interpretability layer that links AI prediction outputs to physical signatures of the underlying energy landscapes. The correspondence between PLM prediction patterns and frustration classes is consistent with the idea that the performance gap arises from differences in energetic embedding. By moving away from traditional internal AI attributions and instead pairing sequence‐space predictions with independent structural energy landscapes, this study offers a robust dual‐stream AI framework for biophysical interpretation of AI predictions. Rather than treating the performance variations of PLMs as simple prediction errors, this dual‐stream approach leverages these variations as diagnostic indicators of a protein's underlying evolutionary and biophysical organization.

PLMs are trained by optimizing an amino acid masking objective across millions of diverse sequences, allowing them to internalize the statistical consensus and coevolutionary rules that govern protein structure and function. The proposed complementary frustration analysis reveals that these sequence‐learned rules can be intimately associated with the energetic topography of the protein landscape. Indeed, in catalytic kinase regions such as the ATP‐binding cleft, the purifying selection required to preserve phosphotransfer chemistry is associated with the maintenance of a funneled, minimally frustrated local energy landscape. Because these stabilizing interaction networks are highly conserved across the entire kinase superfamily, they present a strong memorization signature in sequence space that the PLM can easily detect with high precision and recall. Allosteric regulatory sites, however, operate under completely different biophysical and evolutionary constraints, which may be partly associated with their low detectability in sequence‐based PLM approaches. Our independent biophysical stream reveals that the residues forming allosteric binding sites are natively embedded within a high‐density network of neutral frustration. While catalytic sites require structural rigidity to align chemical groups for catalysis, allosteric sites must maintain conformational and evolutionary plasticity. The granular subpocket analysis of allosteric regions showed the reduced PLM performance at these sites is not a statistical artifact of label pooling, nor is it an uncorrected error in model training. Instead, the lower detection rate at allosteric sites may reflect an intrinsic feature of the sequence embeddings, which is consistent with a biological requirement for local flexibility and structural transitions.

To preserve this essential flexibility, evolution utilizes a strategy we describe as evolutionary sandboxing. Because the local energy landscape is neutrally frustrated, these regulatory pockets tolerate high sequence drift and mutational variability without destabilizing the global fold of the KD. This may allow allosteric sites to evolve into versatile regulatory hubs that can accommodate diverse chemical ligands and produce different functional outcomes. When a sequence‐trained PLM processes an allosteric region, it encounters a variable, low‐consensus sequence signature that is statistically indistinguishable from the generic structural background. Our findings are also consistent with the pioneering DMS studies (Beltran et al., 2026; Escobedo et al., 2025; Faure et al., 2022; Hidalgo‐Carcedo et al., 2026) showing that allosteric interactions and allosteric hotspots are widespread, distributed, and frequently mediated by distal, surface‐exposed regions. According to these studies, regulatory sites can often tolerate substitutions while still modulating function, which is a pattern that aligns with the neutral frustration signature we observe at allosteric sites.

Noteworthy, the correlation between frustration class and detectability does not imply causation as other factors such as class imbalances along with training data size, compositions, and diversity may significantly affect PLM performance. While the frustration analysis provides a plausible and complementary physical interpretation, causality cannot be inferred from these analyses alone. Nonetheless, the proposed dual‐stream framework underscores that physics‐based energy landscapes and local frustration analysis can be viewed as powerful complementary tools for understanding and interpreting the performance of AI models.

Sequence‐trained models provide an exceptional tool for rapid, high‐throughput functional scanning across the entire human kinome, highlighting regions of high evolutionary constraint. When these models encounter a predictability gap, such as the sharp split between orthosteric and allosteric spaces, the independent physics‐based frustration stream provides the necessary interpretive tool to decode the structural properties of that site. Overcoming the allosteric blind spot may also benefit from incorporating energy landscape signatures and frustration indices as natural priors: for example, regularization terms penalizing overconfidence in neutrally frustrated zones, as edge weights in graph neural networks to capture allosteric couplings, or as auxiliary targets in multi‐task learning frameworks.

More broadly, the proposed dual‐stream strategy can be generalized to other protein families and other functional sites (e.g., protein–protein interaction interfaces, cryptic pockets, and post‐translational modification sites). By aligning data‐driven learning with energy landscape theory, predictive AI models can be transformed into more robust tools for mechanistic discovery, enabling a deeper probing and understanding of how evolution encodes regulatory complexity in proteins.

## 4 CONCLUSIONS

The proposed integration of PLM predictions and energy landscape theory offers a lens through which to view the regulatory architecture of the human kinome. Converting the algorithmic limitations of AI models into a diagnostic signal suggests that the allosteric blind spot may reflect intrinsic biophysical properties of regulatory sites rather than purely technical limitations. Across a rigorously curated dataset of human kinase–ligand complexes, we observe a consistent dichotomy: orthosteric ATP‐binding sites are detected with high precision by sequence‐based models, while allosteric sites evade detection despite preserved ranking ability. This performance gap correlates with frustration class, which is consistent with the hypothesis that energy landscape organization influences algorithmic visibility. Our results suggest that within the kinase family minimally frustrated regions can encode stable, evolutionarily conserved interactions that give rise to strong sequence signals, while neutral frustration is associated with allosteric versatility and regulatory diversity that lack consistent evolutionary signatures. In this context, prediction confidence can be understood as a readout of how strongly functional constraints are embedded in sequence space. Rather than viewing these differences as model deficiencies, our results position them as diagnostic signals that expose the biophysical logic of protein regulation.

This perspective reframes PLM predictions combined with the frustration analysis as a robust framework for probing the relationship between sequence, structure, and energetics, enabling systematic identification of regions where functional roles are not directly encoded in evolutionary conservation. By demonstrating that algorithmic visibility is governed by energetic frustration, we establish a quantitative baseline against which future physics‐informed models can be calibrated.

As high‐quality structural and mutational data continue to accumulate across protein families, the principles elucidated here may inform the development of interpretable, mechanistically grounded AI tools. This study highlights the importance of aligning AI approaches with the physical principles governing biomolecular systems, providing a foundation for the development of interpretable and mechanistically grounded AI frameworks. More broadly, this work outlines an explainable AI strategy for bridging data‐driven learning with energy landscape theory, transforming predictive models into tools for probing biological phenomena and mechanistic inference, enabling a deeper understanding of how evolution encodes regulatory complexity and allostery in proteins. Ultimately, this framework may provide a high‐throughput diagnostic instrument for the rational engineering of allosteric modulators, moving us closer to a unified theory of protein allostery.

## 5 MATERIALS AND METHODS

### 5.1 Dataset curation and evaluation framework

To ensure rigorous evaluation of model generalization and avoid potential train–test leakage, we implemented a sequence similarity filtering strategy between training and evaluation datasets. The PLM was trained on a subset of the LIGYSIS dataset (3006 sequences). To eliminate overlap with the kinase dataset used for evaluation, all training sequences sharing greater than 30% sequence identity with any protein in the KinCoRe dataset were removed using MMseqs2 clustering (Kallenborn et al., 2025; Mirdita et al., 2019; Steinegger & Söding, 2017). No representative selection was performed; any sequence exceeding the similarity threshold was excluded. This filtering resulted in a reduced training set of 2949 sequences (denoted LIGYSIS_SI30), ensuring that all reported results reflect genuine generalization rather than memorization. The evaluation dataset was derived from the KinCoRe database (Faezov & Dunbrack Jr., 2023; Gizzio et al., 2026; Modi & Dunbrack Jr., 2019, 2022) yielding a total of 9901 kinase–ligand complexes (structure–chain pairs) spanning 453 human kinases (Table 1). The dataset includes five mechanistic classes of inhibitor binding defined by DFG motif conformation and αC‐helix position: Types I, I.5, II (orthosteric), and Types III and IV (allosteric). Because structural datasets are inherently imbalanced with certain kinases being overrepresented (Tables 2 and 3), we constructed a non‐redundant subset (KinCoRe‐UNQ) (Riedlová et al., 2026) by selecting one structure per UniProt accession (708 complexes). This controls for biases arising from repeated structures of identical or highly similar sequences. Comparative analysis between the full dataset and KinCoRe‐UNQ demonstrated minimal differences in model performance, indicating that results are not driven by redundancy. Importantly, the dataset exhibits a strong class imbalance, with orthosteric sites (Types I, I.5, and II) comprising ~90% of all complexes, while allosteric sites (Types III and IV) account for only ~7%.

### 5.2 Ground‐truth binding residue mapping, spatial discontinuity, and sensitivity validation

To ensure absolute objectivity and eliminate any potential investigator bias in defining binding site boundaries, a completely automated and deterministic coordinate‐geometry pipeline was established. The fine‐tuned PLM operates entirely at the single‐residue level, receiving only the primary sequence as input and computing an independent, continuous probability score for each amino acid position without any prior knowledge of pocket locations or ligand identities. Consequently, the assignment of ground‐truth labels is performed purely as an unbiased evaluation step based on the corresponding experimental three‐dimensional coordinates from the KinCoRe database.

For the baseline model configuration, a ground‐truth binary label of binding residue was assigned to a residue if any of its heavy atoms resided within a distance threshold from any heavy atom of the co‐crystallized or resolved ligand; otherwise, non‐binding background. This explicit residue‐level mapping circumvents the structural ambiguity of assigning rigid macroscopic “pocket boundaries” and directly captures the physical contact surface. To address the inherent spatial discontinuity of allosteric pockets and their tendency to engage non‐sequential structural elements, this mapping mechanism operates over the entire primary sequence globally, seamlessly incorporating discontinuous residues that converge in three‐dimensional space to form the functional binding interface.

To rigorously validate that the observed hierarchy of PLM detectability—and specifically the poor performance observed for distal allosteric sites—is a robust biophysical phenomenon rather than an artifact of an arbitrary distance boundary, a multi‐tiered sensitivity and robustness analysis was executed. The heavy‐atom distance threshold cutoff was systematically varied across four configurations: strict core contacts, baseline definition, expanded contact envelope, and permissive pocket‐lining boundary. For each distance regime, the complete set of ground‐truth residue labels was regenerated dynamically across all 453 kinase structures, and all threshold‐free (AUROC and AUPR) and threshold‐dependent (MCC, F1 score, and Accuracy) performance metrics were recomputed globally and per inhibitor class. To systematically assess the robustness of our conclusions to these ambiguities, we performed several complementary analyses that vary the labeling criteria. We repeated labeling using distance cutoffs of 3.0, 4.0 (primary), 5.0, and 6.0 Å. This tests sensitivity to strict versus liberal contact definitions.

For ABL kinase, we selected five distinct conformational states (active, inactive, Asciminib‐bound, GNF‐2‐bound, and DPH‐bound). For each residue, we asked whether it contacts any allosteric ligand (4.0 Å cutoff) in any of the five structures. The consensus set across conformations represents the “conformationally invariant” binding pocket. We compared PLM performance on this ensemble‐defined set versus single‐structure definitions.

We also employed soft (distance‐weighted) probabilistic labels. Instead of a hard binary label, we assigned each residue a continuous binding probability based on its minimal distance to any ligand heavy atom: $p_{\text{bind}}=\exp\left(-d_{\min}/2\right)$ for $d_{\min}\leq 5.0\;Å$, and 0 otherwise. This function decays from 1 (at $d_{\min}=0$) to ~0.08 at 5.0 Å. We then computed threshold‐free metrics (AUROC and AUPR) using these soft labels as ground truth, which avoids arbitrary discretization.

Allosteric regulation in protein kinases frequently involves highly dynamic and conformationally plastic structural elements, most notably the activation loop (A‐loop) and the C‐helix. To ensure that the diffuse and lower‐confidence PLM predictions at allosteric pockets were not artificially driven by localized coordinate ambiguity or structural mobility in these specific motifs, a dynamic segment exclusion profiling protocol was implemented. In this protocol, all residues mapping to the canonical C‐helix and the activation loop (defined via standardized KinCoRe alignment profiles) were completely masked out and excluded from the performance evaluation matrix, allowing performance to be evaluated solely on the remaining structured regions.

All performance metrics (AUROC, AUPR, MCC, F1, and accuracy) were recomputed under each labeling definition.

### 5.3 Sub‐classification of binding sites by pocket anatomy and inhibitor scaffold

To test whether the observed performance gap between orthosteric and allosteric sites could be explained by label heterogeneity within the broad KinCoRe classes, we performed a detailed sub‐classification of all binding sites based on structural anatomy (for allosteric sites) and inhibitor chemical scaffold (for orthosteric sites, as a control). We manually inspected all Types III and IV complexes in the KinCoRe dataset (*n* = 969) and assigned each to one of seven anatomically defined pocket types: (a) myristoyl pocket (C‐lobe, distal), (b) DFG‐out pocket (adjacent to the ATP site, often Type III), (c) αC‐helix pocket, (d) P + 1 loop pocket (activation loop region), (e) C‐lobe groove, (f) N‐lobe pocket, and (g) other (<5 complexes). Assignment was based on the published pocket coordinates and verified by structural overlay using the conserved kinase core as reference. For each subtype with at least 10 complexes, we recomputed PLM performance metrics (AUROC, AUPR, and MCC) and frustration densities.

To eliminate any remaining heterogeneity, we assembled a dedicated test set of the ABL myristoyl pocket, the best‐characterized distal allosteric site. We collected 14 ABL structures (isolated KD and SH3–SH2–KD assemblies) bound to various myristoyl pocket ligands (Asciminib, GNF‐2, DPH, myristic acid, Imatinib in resistant variants). All structures were aligned to the conserved kinase core, and the myristoyl pocket residues were defined by the union of residues within 4.0 Å of any myristoyl‐binding ligand across all ABL structures. This yielded a consensus pocket of 43 residues. For all sub‐classification analyses, we used the Mann–Whitney *U* test to compare performance distributions between subtypes and against the original aggregated classes. Frustration densities were compared using the same test.

### 5.4 Protein representation and model architecture

Protein sequences were encoded using the ESM‐2 (650 M) PLM (Lin et al., 2023), a transformer‐based architecture trained on large‐scale sequence data to capture evolutionary and contextual relationships. In this work, the model is not treated solely as a predictive tool, but as a diagnostic probe of sequence‐encoded biophysical information, where prediction performance reflects the degree to which functional sites are evolutionarily constrained and therefore “visible” in sequence space. For each sequence, residue‐level embeddings (1280 dimensions) were extracted and used as input features. These embeddings encode both local physicochemical context and long‐range sequence dependencies, enabling structure‐aware inference without explicit structural input. In this framework, PLMs are not treated as end‐point predictors but as diagnostic probes of sequence‐encoded biophysical information. A lightweight classification head was applied to map residue embeddings to binding‐site probabilities (Riedlová et al., 2026). This design preserves the underlying evolutionary representation while enabling task‐specific interpretation.

We implemented and adapted a previously introduced two‐stage hierarchical fine‐tuning protocol (Riedlová et al., 2026). In Phase I (head‐only training), the transformer encoder was frozen, and only the classification head parameters were optimized for two epochs. During Phase II (end‐to‐end fine‐tuning), all model parameters were unfrozen and jointly optimized for one additional epoch, allowing adaptation to kinase‐specific patterns while maintaining global sequence features (Riedlová et al., 2026).

### 5.5 Prediction and evaluation framework

The model outputs residue‐level probabilities $p_{i}\in \left[0,1\right]$, which were evaluated using both threshold‐free and threshold‐dependent metrics. Threshold‐free performance was assessed using AUROC and AUPR. For binary classification, two thresholding strategies were used: Fixed threshold (PLM‐fixed) with a global threshold (*t* = 0.75) selected by maximizing Matthews correlation coefficient (MCC) on validation data and class‐optimized threshold (PLM‐bestMCC) where thresholds were optimized separately for each inhibitor class via grid search (*t* ∈ [0,1], step 0.05), accounting for differences in evolutionary constraint between orthosteric and allosteric sites. Binary predictions were evaluated using MCC, F1 score, and accuracy. Analyses were performed at both micro‐averaged (global) and macro‐averaged (per‐structure) levels to account for class imbalance and structural heterogeneity.

### 5.6 Structural analysis and preparation of kinase structures

To establish a direct correspondence between sequence‐based predictions and the physical organization of kinase structures, we constructed a structurally standardized ensemble of KDs spanning major families, conformational states, and inhibitor classes represented in the KinCoRe dataset. This step was essential to ensure that both prediction outputs and energetic analyses are interpreted within a consistent structural and biophysical framework. Representative structures were selected from the PDB based on stringent criteria, including high crystallographic resolution (≤3.0 Å), coverage of major kinase groups (A major group of serine/threonine protein kinases named after its primary representative families Protein Kinase A (PKA), Protein Kinase G (PKG), and Protein Kinase C (PKC) [AGC], CAMK: Calcium/calmodulin‐dependent protein kinase group; CMGC: A major kinase group named after its initial members: CDK (Cyclin‐dependent kinase), mitogen‐activated protein kinase [MAPK], glycogen synthase kinase 3 [GSK3], and CDC‐like kinase [CLK]; sterile kinase group [STE], Tyrosine Kinase group [TK]; Tyrosine Kinase‐Like group [TKL]), and representation of diverse inhibitor binding modes (Types I–IV). Selection was guided to preserve the conformational diversity captured by KinCoRe, including active (DFG‐in/αC‐in) and inactive (DFG‐out or αC‐out) states, thereby enabling systematic comparison across orthosteric and allosteric regulatory regimes.

All structures underwent a uniform and rigorous preprocessing pipeline to ensure compatibility with downstream energetic analysis. This included removal of crystallographic artifacts (water molecules, buffer components, and non‐cognate ligands), retention of the biologically relevant inhibitor where appropriate, and reconstruction of missing side chains and unresolved local regions when feasible. All structures were obtained from the PDB (Rose et al., 2017). Hydrogen atoms and missing residues were initially added and assigned according to the WHATIF program web interface (Hekkelman et al., 2010; Hooft et al., 1996). The structures were further pre‐processed through the Protein Preparation Wizard (Schrödinger, LLC, New York, NY) for assignment and adjustment of ionization states, formation of assignment of partial charges as well as an additional check for possible missing atoms and side chains that were not assigned by the WHATIF program. The missing loops in the Cryogenic Electron Microscopy (cryo‐EM) structures were reconstructed using template‐based loop prediction approaches ModLoop (Fiser & Sali, 2003) and ArchPRED (Fernandez‐Fuentes et al., 2006) and further confirmed by FALC (Fragment Assembly and Loop Closure) program (Ko et al., 2011). The side chain rotamers were refined and optimized by the SCWRL4 tool (Krivov et al., 2009).

Prior to frustration analysis, all kinase–ligand complexes were subjected to a multi‐stage structural refinement and energy minimization protocol to resolve steric clashes and optimize hydrogen‐bonding networks. The AMBER22 suite was used for all refinements (Case et al., 2022). Protein atoms were modeled using the ff19SB force field, while ligand parameters were generated via the General Amber Force Field (GAFF2) (He et al., 2020) with AMBER antechamber module (Case et al., 2023; He et al., 2020). To prevent structural distortion, a layered minimization protocol was executed: (a) 1000 steps of steepest descent were performed with all heavy atoms (protein and ligand) restrained, allowing for the optimization of hydrogen atom positions and protonation states; (b) 2000 steps focusing on the ligand and surrounding water molecules, with the protein backbone and side chains held under harmonic restraints; (c) 2000 steps targeting protein side chains to resolve localized atomic overlaps within the protein interior; (d) 2000 steps of backbone‐only refinement to ensure secondary structure stability; (e) a final 5000‐step gradient minimization was performed with all restraints removed. This ensured the complex reached a local potential energy minimum, providing a consistent energetic baseline for the subsequent calculation of configurational and mutational frustration indices. To enable cross‐structure comparison, KDs were structurally aligned using conserved elements of the catalytic core. Specifically, alignment was performed based on Cα atoms of the central β‐sheet and key conserved motifs, including the HRD catalytic loop and the DFG motif. This procedure establishes a common structural reference frame that preserves functional architecture while minimizing variability arising from peripheral regions. This structurally harmonized ensemble provides the foundation for two key analyses. First, it enables consistent mapping of residue‐level prediction probabilities onto three‐dimensional structures, allowing direct visualization of model‐detected binding regions across kinase families. Second, it ensures that local frustration calculations are performed on energetically comparable and physically consistent conformations, which is critical for interpreting frustration signatures in terms of intrinsic energy landscape features rather than structural artifacts.

### 5.7 Local frustration analysis

To characterize the energetic signatures of functional sites, we employed local frustration analysis, a framework rooted in energy landscape theory that quantifies how favorably each native residue–residue interaction is optimized relative to an ensemble of decoy configurations (Ferreiro et al., 2007; Parra et al., 2016). This approach distinguishes residues that are evolutionarily stabilized from those involved in conformational adaptability or functional stress. Two complementary frustration indices were computed for each residue–residue contact in the protein structure. Configurational (conformational) frustration assesses the sensitivity of a native contact to local structural perturbations. It is calculated by randomizing both residue identities and interatomic distances within the native contact geometry, generating an ensemble of 1000 decoy contacts. Mutational frustration evaluates the energetic optimality of a given residue–residue pair by comparing its interaction energy to that of all possible amino acid substitutions at the same positions, while the backbone conformation remains fixed. The same decoy generation procedure (1000 decoys) is used. Both frustration indices were expressed as *Z*‐scores, defined as:

(1) $$Z=\frac{E_{\text{native}}-\left\langle E_{\text{decoys}}\right\rangle }{\sigma \left(E_{\text{decoys}}\right)},$$

where $E_{\text{native}}$ is the energy of the native contact, and $\left\langle E_{\text{decoys}}\right\rangle$ and $\sigma \left(E_{\text{decoys}}\right)$ are the mean and standard deviation of energies computed from the decoy ensemble. Following well‐established thresholds validated across diverse protein systems (Ferreiro et al., 2007; Parra et al., 2016) contacts were classified as below.

Minimally frustrated (Z>0.78): the native interaction is significantly more favorable than decoy alternatives, indicating strong energetic optimization and evolutionary constraint. Neutrally frustrated (−1.0≤Z≤0.78): the native interaction is neither strongly favored nor disfavored, consistent with conformational plasticity and mutational tolerance. Highly frustrated (Z<−1.0): the native interaction is significantly less favorable than decoy alternatives, reflecting local strain or conflict. These thresholds were derived from statistical analysis of protein decoy ensembles and are not family‐specific; they have been successfully applied to a wide range of protein systems, including kinases (Ferreiro et al., 2007; Parra et al., 2016; Raisinghani et al., 2024).

To obtain residue‐level frustration profiles, we computed for each residue the local density of frustrated contacts within a 5 Å radius. Specifically, for a given residue i, we identified all residues j such that the Cα‐Cα distance between them is ≤5 Å. Among these contacts, we counted how many were minimally, neutrally, or highly frustrated. The relative frustration density for a class was defined as the number of contacts in that class divided by the total number of contacts within the 5 Å neighborhood. A residue was assigned a dominant frustration category if more than 50% of its interacting contacts within the most populated conformational ensemble belonged to the same frustration class. The frustration analysis proceeded as follows: (a) For each protein structure in the KinCoRe dataset, we extracted all residue–residue contacts with Cα‐Cα distance ≤10 Å (the default cutoff for frustration calculation); (b) for each contact, configurational and mutational *Z*‐scores were computed using the frustratometeR R package (version 2.0, https://github.com/proteinphysiologylab/frustratometeR); (c) each contact was classified into one of the three frustration regimes using the *Z*‐score thresholds above; (d) contact‐level classifications were converted to residue‐level densities by counting contacts within a 5 Å radius; and (e) we assigned a dominant frustration category to each residue using the 50% majority rule. All calculations were performed on a high‐performance computing cluster using the supercomputer implementation of frustratometeR. The complete analysis pipeline is also available in the public GitHub repository (https://github.com/kiarka7/plm-vs-p2rank-kinases).

### 5.8 Statistical analysis

Statistical significance of performance differences between the PLM and baseline methods was determined using paired two‐tailed *t*‐tests, with *p* < 0.05 considered significant. For multi‐family comparisons (e.g., across five kinase types), *p*‐values were adjusted via the Benjamini–Hochberg false discovery rate (FDR) correction (Benjamini & Hochberg, 1995). To ensure a balanced interpretation under class‐imbalanced conditions, both micro‐ and macro‐averaging schemes were employed. Micro‐averaged metrics were computed globally across all residues, while macro‐averaged metrics were calculated per structure and then averaged across the dataset, giving equal weight to each kinase irrespective of chain length or residue count. For the frustration analysis, residue‐level frustration densities were compared across orthosteric and allosteric sites using the Mann–Whitney *U* test, a non‐parametric method appropriate for non‐normal distributions and unequal variances (Chicco et al., 2025). All statistical computations were performed in Python 3.10 using SciPy (v1.10) for hypothesis testing, statsmodels (v0.14) for multiple comparison correction, and custom scripts for effect size and correlation analysis. Visualization of distributions and significance annotations were generated with Matplotlib (v3.7) and Seaborn (v0.12).

## AUTHOR CONTRIBUTIONS

Conceptualization: **GMV**; Methodology: **WG**, **ML**, **LT**, **BF**, **KR**, **VS**, **MN**, **DH**, **GMV**; Software: **WG**, **ML**, **LT**, **BF**, **KR**, **VS, MN**, **DH**, **GMV**; Validation: **WG**, **ML**, **LT**, **BF**, **KR**, **VS**, **DH**, **GMV**; Formal analysis, **WG**, **ML**, **LT**, **BF**, **KR**, **VS**, **DH**, **GMV**; Resources: **WG**, **ML**, **LT**, **BF**, **KR**, **VS**, **DH**, **GMV**; Data curation: **WG**, **ML**, **LT**, **BF**, **KR**, **VS**, **DH**, **GMV**; Writing‐original draft preparation: **KR**, **VS**, **DH**, **GMV**; Writing‐review and editing: **KR**, **VS**, **DH**, **GMV**; Visualization: **WG**, **ML**, **LT**, **BF**, **KR**, **VS**, **MN**, **DH**, **GMV**; Supervision: **DH**, **GMV**; Project administration: **DH**, **GMV**; Funding acquisition: **DH**, **GMV**. All authors have read and agreed to their individual contributions. All authors have approved the submission of this manuscript.

## CONFLICT OF INTEREST STATEMENT

The authors declare no conflict of interest. The funders had no role in the design of the study; in the collection, analyses, or interpretation of data; in the writing of the manuscript; or in the decision to publish the results.

## Supporting information


**Data S1.** This document contains nine supplementary figures (Figures S1–S9) providing detailed computational benchmarks, architectural schematics, and extended biophysical profiles. The document also includes nine supplementary tables (Tables S1–S9) providing detailed statistics, robustness model analysis, and sensitivity analysis of PLM metrics across various binding sites partition and subtypes definitions.

## Data Availability

Data for this article, including description of data types, all data, software and scripts are freely available at the Github repository https://github.com/kiarka7/plm-vs-p2rank-kinases and the Zenodo archive (https://doi.org/10.5281/zenodo.18226590). The fine‐tuned PLM model can be downloaded from this data storage: https://owncloud.cesnet.cz/index.php/s/8RSJqt60D2uWJNa. The code and datasets related to this protein language model (PLM) are available at: https://github.com/skrhakv/LBS-pLM. The Github repository https://github.com/kiarka7/plm-vs-p2rank-kinases contains publicly available scripts required to reproduce PLM predictions reported in the study. Inference was performed using custom Python scripts that wrap the fine‐tuned ESM2 checkpoint and implement residue extraction, tokenization, batching, and model evaluation. All scripts required to reproduce the PLM predictions reported here are publicly available in GitHub repository (https://github.com/kiarka7/plm-vs-p2rank-kinases). The fine‐tuned model checkpoint can be provided upon request for reproducibility. Local protein frustration and energy landscape analyses were performed using a supercomputer implementation of the frustratometeR R package (https://github.com/proteinphysiologylab/frustratometeR). This implementation was utilized to calculate energetic local frustration, assess the effects of amino acid variants, and analyze frustration across molecular dynamics simulations. Zenodo archive (https://doi.org/10.5281/zenodo.18226590) provides the datasets, processed prediction outputs, evaluation results, figures, and scripts used in the study. The archive mirrors the directory structure expected by the analysis pipeline and contains two dataset variants: (a) KinCoRe, which includes all protein–ligand complexes used for the main analyses reported in the manuscript and (b) KinCoRe‐UNQ, a reduced variant retaining a single representative structure per protein (UniProt ID), which was used for Supporting Information S1 analyses to control for over‐representation of proteins with multiple solved structures. All results presented in the manuscript are derived from the KinCoRe dataset, while KinCoRe‐UNQ is used for analyses reported in Supporting Information S1. Protein structures are not included in this archive. Crystal structures were obtained and downloaded from the Protein Data Bank (http://www.rcsb.org). The rendering of protein structures was done with the UCSF ChimeraX package (https://www.rbvi.ucsf.edu/chimerax/) and Pymol (https://pymol.org/2/).
