# Supplementary material for: Decoding the allosteric grammar of protein kinases: A dual‐stream framework integrating protein language models and energy landscape frustration analysis
Source: Protein Sci. 2026 Jul 9;35(8):e70714. doi: 10.1002/pro.70714 (PMC13347374; doi:10.1002/pro.70714)
Supplement: Supplementary file 1 — Data S1. This document contains nine supplementary figures (Figures S1–S9) providing detailed computational benchmarks, architectural schematics, and extended biophysical profiles. The document also includes nine supplementary tables (Tables S1–S9) providing detailed statistics, robustness model analysis, and sensitivity analysis of PLM metrics across various binding sites partition and subtypes definitions. [file PRO-35-e70714-s001.pdf]

Supplemental Materials

Decoding the Allosteric Grammar of Protein Kinases: A Dual-Stream Framework Integrating Protein Language Models and Energy Landscape Frustration Analysis

Will Gatlin[1], Max Ludwick[1], Lucas Turano[1], Brandon Foley[1], Kamila Riedlová[2], Vít Škrhák[2], Marian Novotný[3], David Hoksza[2], Gennady M. Verkhivker[1,4,5*]

[1]Keck Center for Science and Engineering, Department of Biological Sciences, Schmid College of Science and Technology, Chapman University, Orange, CA 92866, United States of America

[2]Department of Software Engineering, Faculty of Mathematics and Physics, Charles University, Prague, Czech Republic

[3]Department of Cell Biology, Faculty of Science, Charles University, Prague, Czech Republic

[4]Department of Biomedical and Pharmaceutical Sciences, Chapman University School of Pharmacy, Irvine, CA 92618, United States of America

[5]Department of Pharmacology, Skaggs School of Pharmacy and Pharmaceutical Sciences, University of California San Diego, 9500 Gilman Drive, La Jolla, CA 92093, United States of America

* Correspondence: verkhivk@chapman.edu; Tel.: +1-714-516-4586 (G.V)

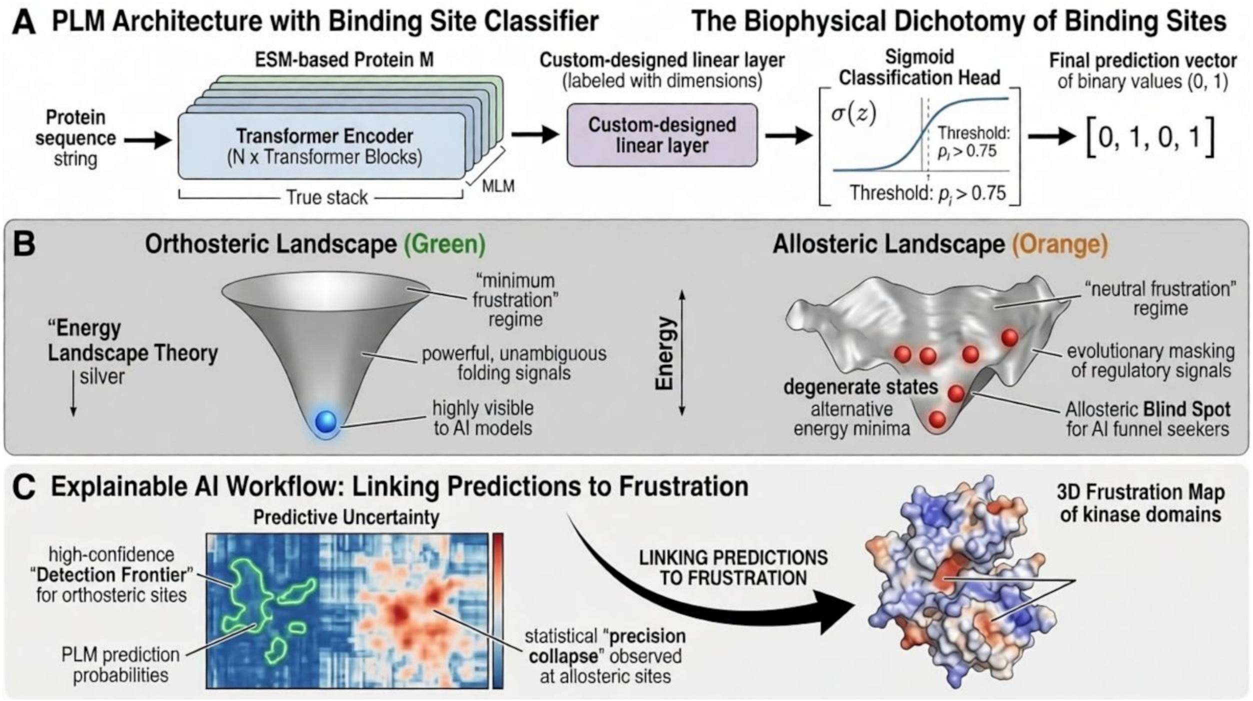

Figure S1. Overview of the dual-stream AI framework integrating PLM-based binding site prediction with the energy landscape frustration analysis. (A) Architecture of the fine-tuned protein language model (PLM) for residue-level binding site classification. A protein sequence is processed by the ESM2-650M transformer encoder, which outputs contextualized residue embeddings. These are passed through a custom-designed linear layer followed by a sigmoid activation to produce per-residue probabilities p_i. A decision threshold ($p_i > 0.75$) converts probabilities to binary predictions, yielding a final prediction vector of binding site residues. (B) Conceptual energy landscapes contrasting orthosteric (green) and allosteric (orange) binding regimes. Orthosteric sites occupy a deep, funneled landscape characterized by minimal frustration, which generates strong, unambiguous folding and binding signals that are highly detectable by AI models. Allosteric sites reside in a shallow, rugged landscape dominated by neutral frustration, where evolutionary masking of regulatory signals creates an "allosteric blind spot" for AI funnel

seekers. (C) Explainable AI/dual-stream component of the workflow linking predictive performance to frustration analysis. High-confidence predictions define a “detection frontier” for orthosteric sites, while allosteric sites exhibit a statistical precision collapse. PLM prediction probabilities are overlaid with 3D frustration maps of kinase domains, enabling a physical interpretation of model behavior through the lens of energy landscape theory.

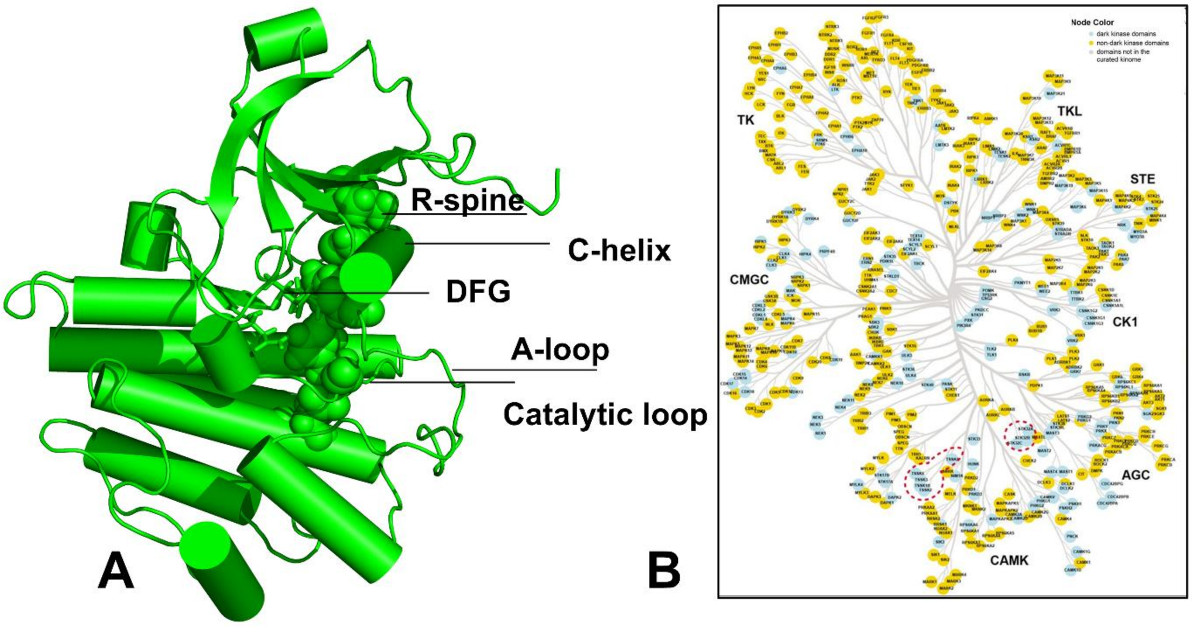

Figure S2. Structural motifs of the kinase catalytic domain and kinome-wide distribution of study targets. (A) Representative 3D structural model of the conserved kinase fold highlighting the regulatory architecture. The Regulatory spine (R-spine) is shown in a vertically aligned, "assembled" state, a hallmark of the active conformation. The DFG motif (Asp-Phe-Gly) is positioned at the start of the activation loop (A-loop), acting as a critical conformational switch; its orientation (DFG-in vs. DFG-out) coordinates with the position of the C-helix to modulate the ATP-binding pocket's volume and energetic accessibility. The catalytic loop provides the necessary residues for phosphotransfer. (B) Phylogenetic tree of the human kinome[64] illustrating the breadth of the current study. Node colors categorize kinases into dark kinase domains. non-dark kinase domains and uncharacterized/non-curated domains. Kinases are obtained from the curated kinome that are visualized on the Coral kinase dendrogram.[65] The recomputed dark kinome is shown in blue and non-dark kinases are shown in yellow. The panel was adopted from a recent study of kinome.[66]

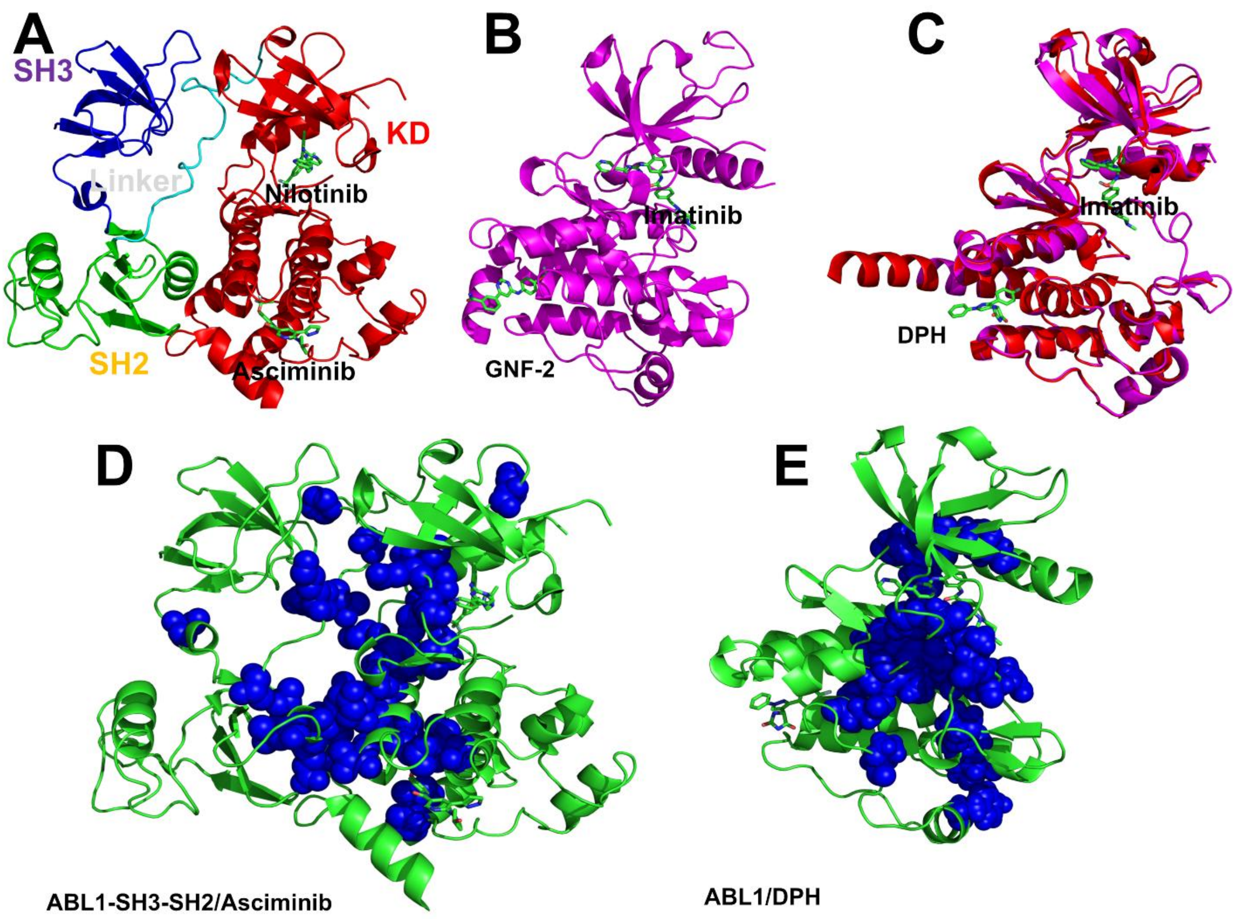

Figure S3. Structural organization of ABL complexes with allosteric modulators and mapping of communication pathways. (A) Architecture of the autoinhibited ABL-SH3-SH2-KD assembly. Crystal structure of the ABL regulatory core in complex with the orthosteric inhibitor Nilotinib and the allosteric inhibitor Asciminib. The multi-domain organization is indicated: SH3 domain (dark blue), SH2 domain (green), SH2-kinase linker (cyan), and the kinase domain (KD, red). Inhibitors are shown in sticks with atom-based coloring. (B) Inactive ABL-KD conformation. Structure of the isolated kinase domain (magenta) in complex with the type II inhibitor Imatinib and the allosteric myristoyl-site inhibitor GNF-2. (C) Active-like ABL-KD conformation. Structural overlay illustrating the ABL-KD in complex with Imatinib and the

allosteric activator DPH. (D–E) Spatial arrangement of allosteric communication networks identified in our previous studies[67] that connect the allosteric myristoyl site and the orthosteric ATP-binding site. (D) Representative connectivity in the ABL1-SH3-SH2/Asciminib complex. (E) Preferential routes in the ABL1/DPH complex. Blue spheres represent the optimal pathways connecting the regulatory myristoyl site with the catalytic machinery.

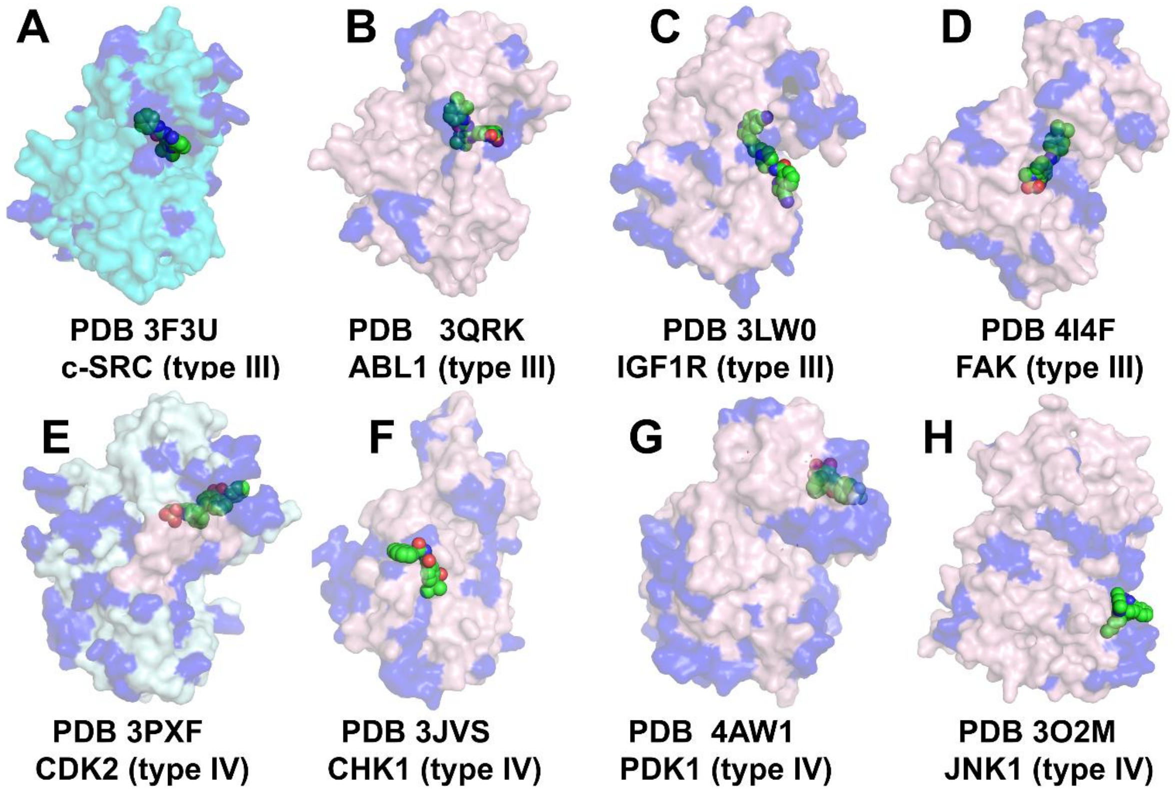

Figure S4. Structural mapping of high neutral frustration density across kinase-allosteric inhibitor complexes. Mapping the regions of high neutral frustration density >0.7 (shown in blue) onto diverse kinase structures with type III and type IV allosteric inhibitors. Protein molecular surfaces are colored by structural origin: pale pink (Chain A) or cyan (Chain B). The corresponding allosteric inhibitors (sphere representation, atom-based coloring) are shown encompassing both Type III (adjacent to the ATP site) and Type IV (distal) modes of allosteric modulation. (Top Row) Type III adjacent-pocket inhibition. High neutral frustration density is seen localized around the orthosteric C-helix/DFG region and adjacent pockets for c-SRC (pdb 3F3U) , ABL (pdb 3QRK), IGF1R (3LW0) and FAK kinase (pdb 4I4F) (Bottom Row) Distal and Type IV allosteric inhibitors. Neutral frustration density in complexes with Type IV allosteric inhibitors CDK2 (3PXF), CHK1 (pdb 3JVS), PDK1 (4AW1) and JNK1 (pdb 3O2M). These neutrally frustrated patches illustrate the structurally diverse "allosteric blind spots."

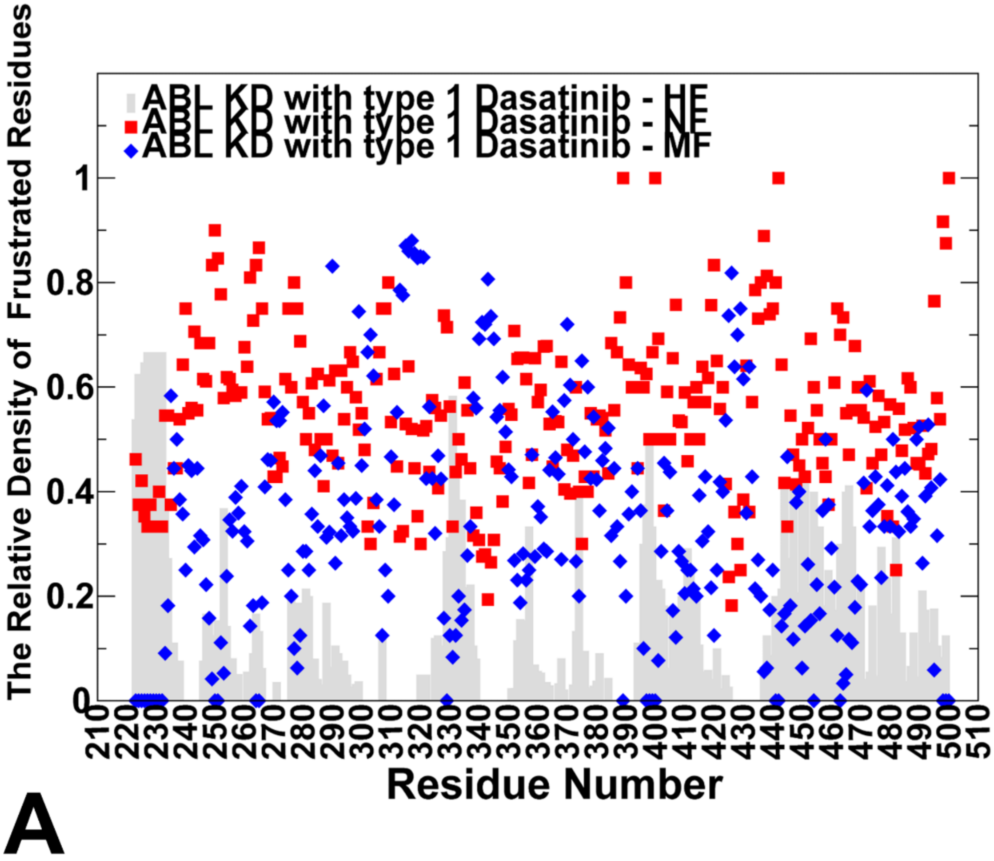
ABL KD with type 1 Dasatinib - HF
ABL KD with type 1 Dasatinib - NF
ABL KD with type 1 Dasatinib - MF
The Relative Density of Frustrated Residues
Residue Number
A

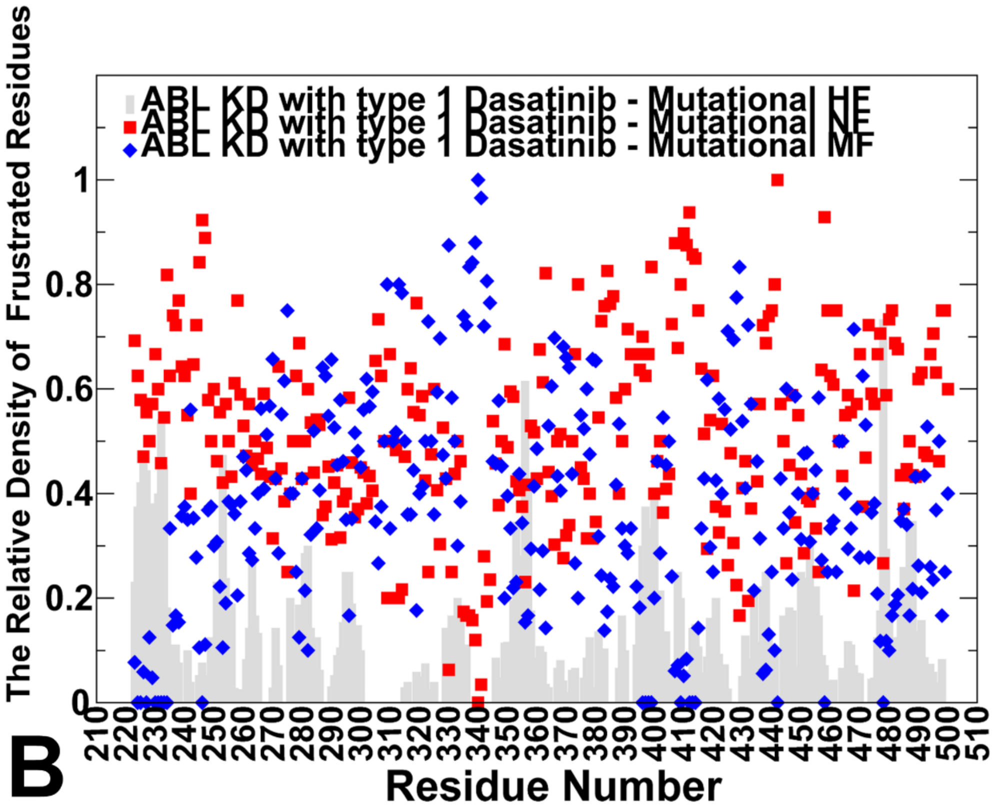
ABL KD with type 1 Dasatinib - Mutational HF
ABL KD with type 1 Dasatinib - Mutational NF
ABL KD with type 1 Dasatinib - Mutational MF
The Relative Density of Frustrated Residues
Residue Number
B

Figure S5. The residue-based distributions of conformational (A) and mutational frustration (B) for ABL complex with type I inhibitors Dasatinib. For all frustration profiles, data are categorized by frustration index: Minimally Frustrated (MF, blue diamonds), Neutrally Frustrated (NF, red squares), and Highly Frustrated (HF, grey bars). Profiles are generated using configurational (spatial) and mutational (evolutionary) decoy sets. (A) Configurational Frustration: The ATP-binding pocket and hydrophobic core exhibit high MF density, indicating structural stabilization. NF and HF residues are restricted to peripheral loops. (B) Mutational Frustration: Strong agreement between MF clusters confirms the orthosteric site is both energetically and evolutionarily optimized, establishing a "locked" baseline.

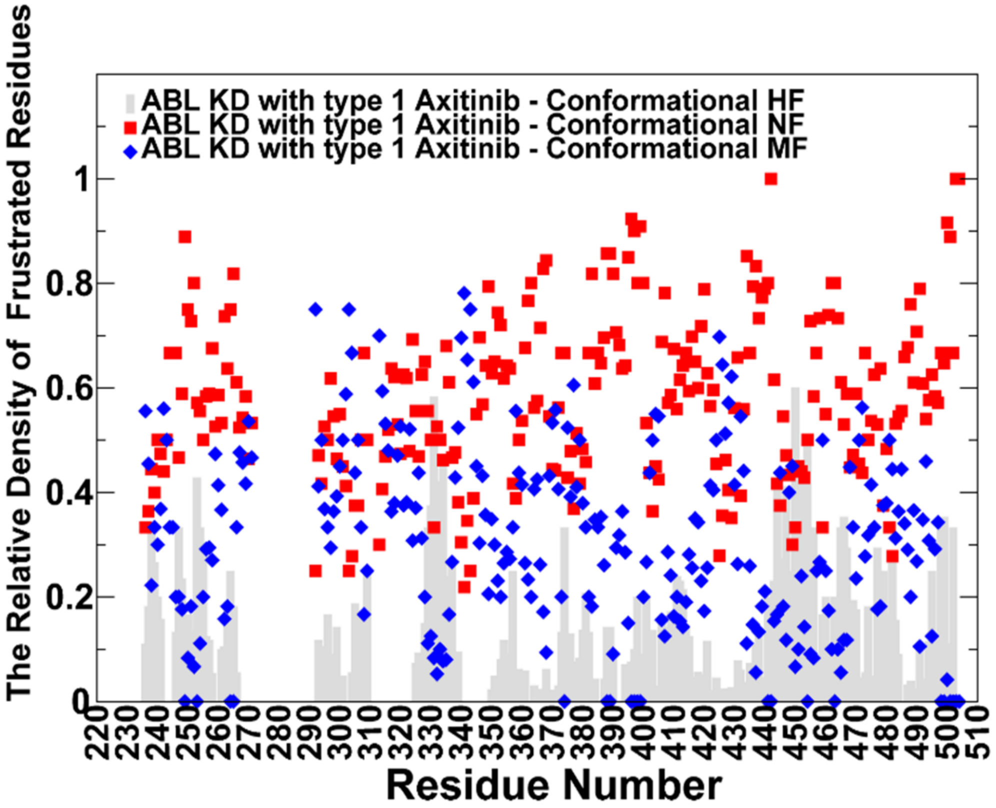
ABL KD with type 1 Axitinib - Conformational HF
ABL KD with type 1 Axitinib - Conformational NF
ABL KD with type 1 Axitinib - Conformational MF
The Relative Density of Frustrated Residues
Residue Number

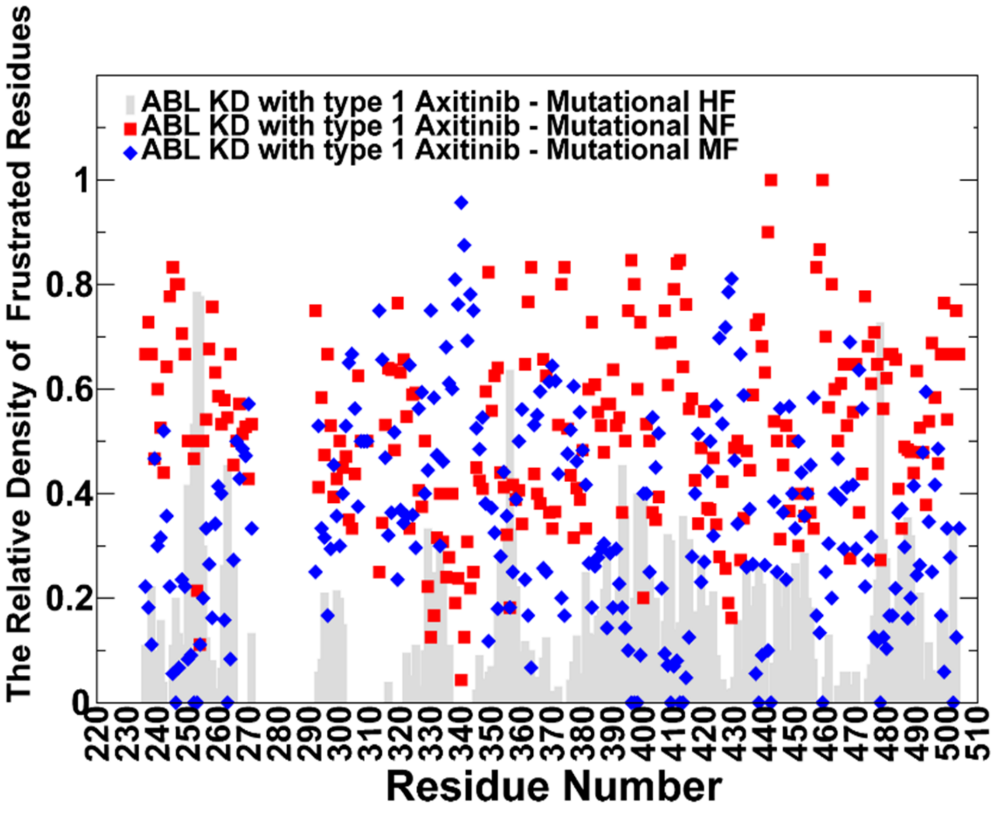
ABL KD with type 1 Axitinib - Mutational HF
ABL KD with type 1 Axitinib - Mutational NF
ABL KD with type 1 Axitinib - Mutational MF
The Relative Density of Frustrated Residues
Residue Number

Figure S6. The residue-based distributions of conformational (A) and mutational frustration (B) for ABL complex with type I inhibitor Axitinib. For all frustration profiles, data are categorized by frustration index: Minimally Frustrated (MF, blue diamonds), Neutrally Frustrated (NF, red squares), and Highly Frustrated (HF, grey bars). Profiles are generated using configurational (spatial) and mutational (evolutionary) decoy sets.

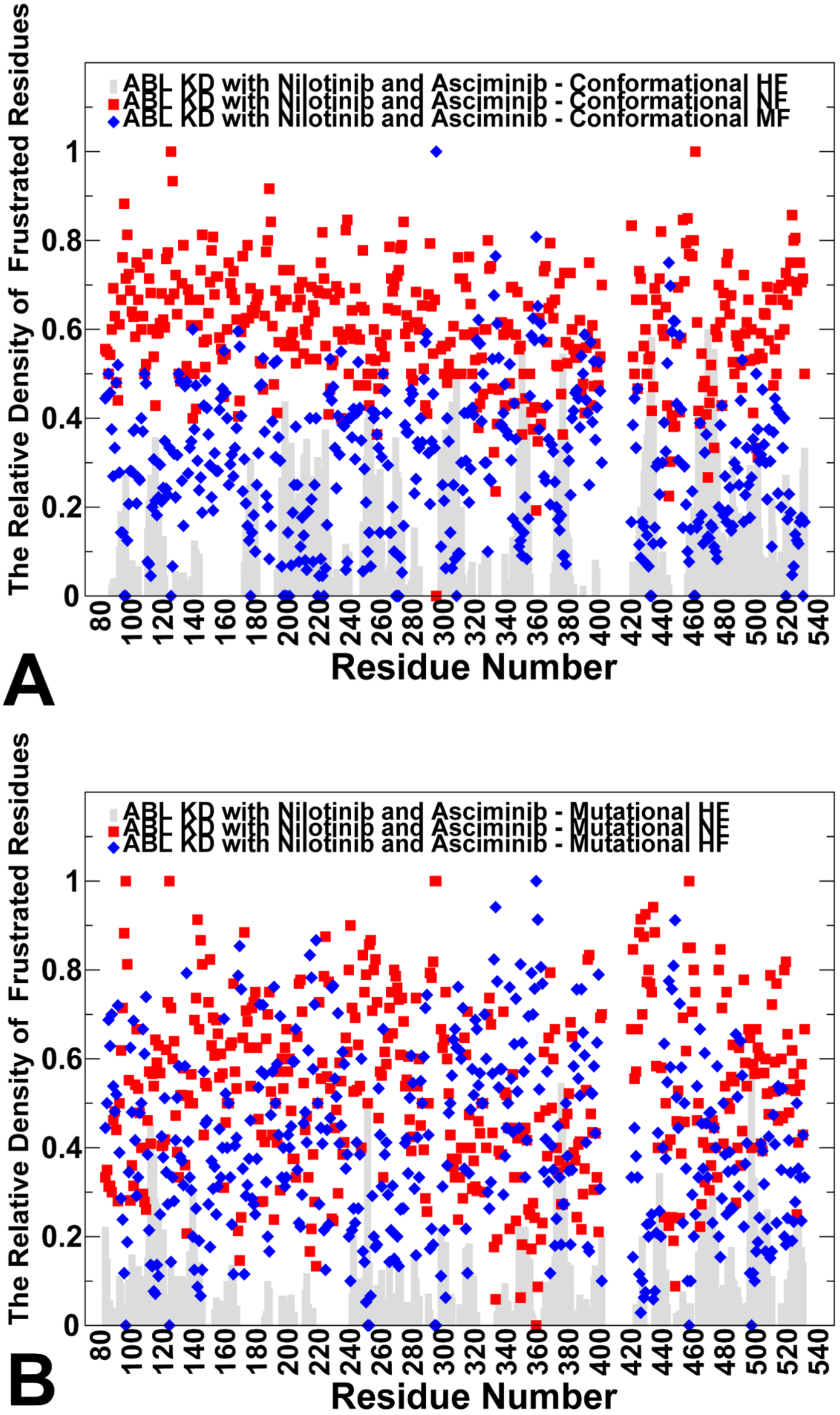
ABL KD with Nilotinib and Asciminib - Conformational HF
ABL KD with Nilotinib and Asciminib - Conformational NF
ABL KD with Nilotinib and Asciminib - Conformational MF
The Relative Density of Frustrated Residues
Residue Number
A
ABL KD with Nilotinib and Asciminib - Mutational HF
ABL KD with Nilotinib and Asciminib - Mutational NF
ABL KD with Nilotinib and Asciminib - Mutational HF
The Relative Density of Frustrated Residues
Residue Number
B

Figure S7. The residue-based distributions of conformational (A) and mutational frustration (B) for the ABL complex with type II inhibitor Nilotinib and allosteric inhibitor Asciminib (pdb id 5MO4). For all frustration profiles, data are categorized by frustration index: Minimally Frustrated (MF, blue diamonds), Neutrally Frustrated (NF, red squares), and Highly Frustrated (HF, grey bars). Profiles are generated using configurational (spatial) and mutational (evolutionary) decoy sets. (A) Configurational Frustration: Widespread MF clusters define the domain interfaces of this "clamped" state (residues 80–540). Notably, the myristoyl pocket remains dominated by NF residues despite Asciminib occupancy. (B) Mutational Frustration: Persistent NF density confirms that the allosteric site maintains a neutral energetic signature independent of ligand-induced stabilization.

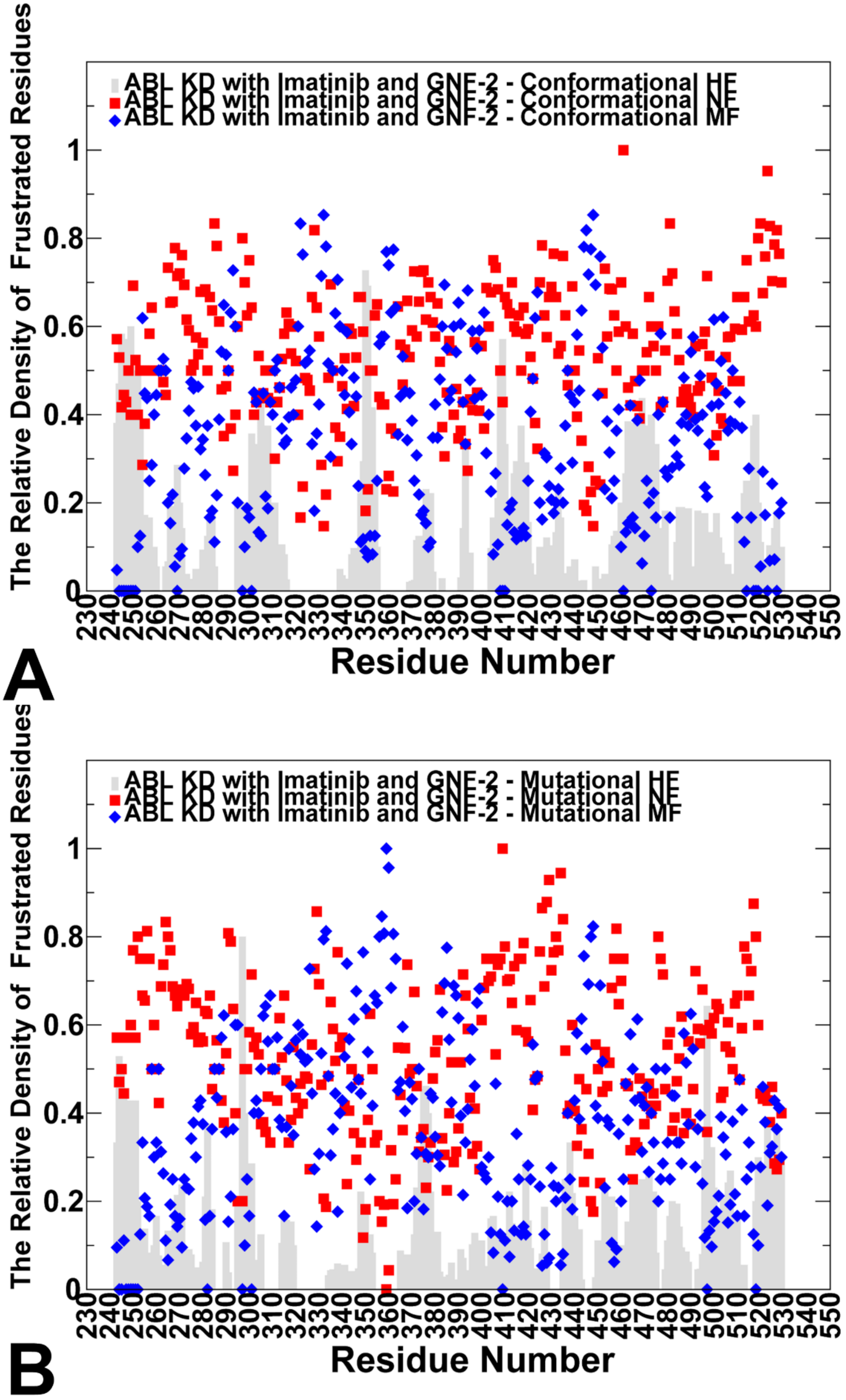
ABL KD with Imatinib and GNF-2 - Conformational HF
ABL KD with Imatinib and GNF-2 - Conformational NF
ABL KD with Imatinib and GNF-2 - Conformational MF
The Relative Density of Frustrated Residues
Residue Number
A
ABL KD with Imatinib and GNF-2 - Mutational HF
ABL KD with Imatinib and GNF-2 - Mutational NF
ABL KD with Imatinib and GNF-2 - Mutational MF
The Relative Density of Frustrated Residues
Residue Number
B

Figure S8. The residue-based distributions of conformational (A) and mutational frustration (B) for the inactive ABL structure bound with type II inhibitor Imatinib and allosteric inhibitor GNF-2 (pdb id 3K5V). For all frustration profiles, data are categorized by frustration index: Minimally Frustrated (MF, blue diamonds), Neutrally Frustrated (NF, red squares), and Highly Frustrated (HF, grey bars). Profiles are generated using configurational (spatial) and mutational (evolutionary) decoy sets. (A) Configurational Frustration: The dual inhibitors stabilize the inactive DFG-out conformation. While the core shows significant MF clusters, the allosteric myristoyl pocket and surrounding network maintain high NF density. (B) Mutational Frustration: Persistent NF density confirms the lack of rigid evolutionary optimization at the allosteric interface.

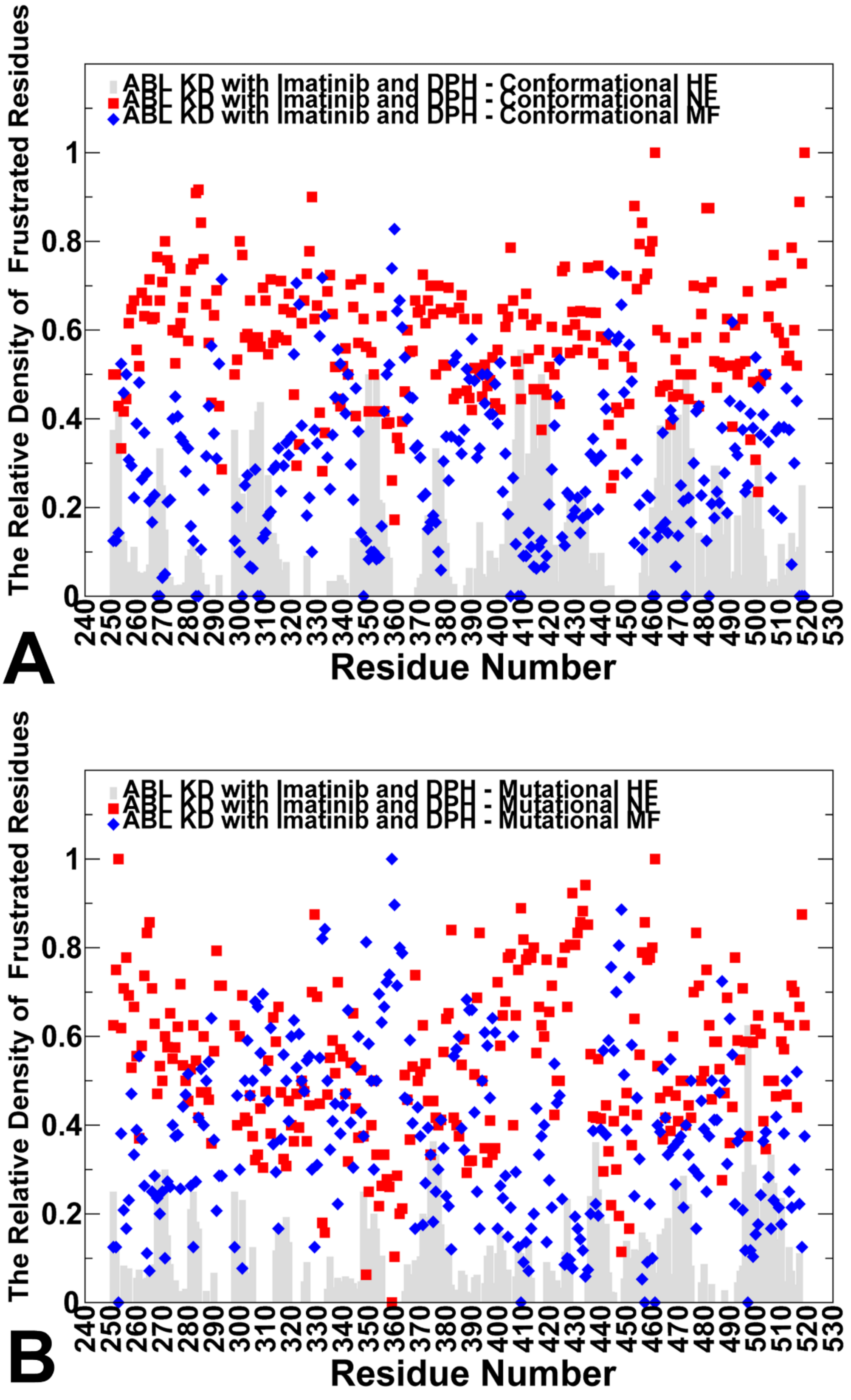
ABL KD with Imatinib and DPH - Conformational HF
ABL KD with Imatinib and DPH - Conformational NF
ABL KD with Imatinib and DPH - Conformational MF
The Relative Density of Frustrated Residues
Residue Number
A
ABL KD with Imatinib and DPH - Mutational HF
ABL KD with Imatinib and DPH - Mutational NF
ABL KD with Imatinib and DPH - Mutational MF
The Relative Density of Frustrated Residues
Residue Number
B

Figure S9. The residue-based distributions of conformational (A) and mutational frustration (B) for the inactive ABL complex with type II Imatinib and allosteric activator DPH (pdb id 3PYY). For all frustration profiles, data are categorized by frustration index: Minimally Frustrated (MF, blue diamonds), Neutrally Frustrated (NF, red squares), and Highly Frustrated (HF, grey bars). Profiles are generated using configurational (spatial) and mutational (evolutionary) decoy sets. (A) Configurational Frustration: Unlike allosteric inhibitors, the activator DPH results in a heterogeneous landscape. While the orthosteric pocket remains stable (MF), the allosteric site exhibits high NF and HF density, reflecting the energetic strain required to disrupt autoinhibition. (B) Mutational Frustration: The allosteric machinery maintains high NF density even under activation, highlighting the shared neutral signature of regulatory pockets.

Table S1. Distribution Statistics for AUROC Across Kinase Site Types.

Site Type	Median	IQR	Range	Distribution Shape
**Orthosteric (Type I/I.5/II)**	0.96–0.98	0.02–0.03	0.92–0.99	Narrow, tall violin; sharp peak near maximum; minimal tail
**Type III Allosteric**	0.88–0.92	0.06–0.08	0.75–0.95	Moderate width; broader than orthosteric
**Type IV Allosteric**	0.65–0.75	0.12–0.15	0.50–0.85	Very broad, flattened; many structures near random (0.5)

Table S2. Distribution Statistics for AUPR Across Kinase Site Types.

Site Type	Median	IQR	Range	Distribution Shape
**Orthosteric (Type I/I.5/II)**	0.65–0.75	0.08–0.10	0.40–0.85	Moderately broad, right-skewed; long tail toward lower values
**Type III Allosteric**	0.25–0.35	0.15–0.20	0.10–0.60	Very broad, nearly flat; dramatic spread
**Type IV Allosteric**	0.05–0.12	0.08–0.12	0.02–0.25	Extremely broad, collapsed near zero; nearly flat

Table S3. Distribution Statistics for MCC Across Kinase Site Types.

Site Type	Median	IQR	Range	Distribution Shape
**Orthosteric (Type I/I.5/II)**	0.58–0.69	0.05–0.07	0.40–0.75	Narrow, tall violin; sharp peak in 0.6–0.7 range
**Type III Allosteric**	0.38–0.45	0.10–0.12	0.15–0.60	Moderately broad, nearly symmetric
**Type IV Allosteric**	0.05–0.15	0.12–0.15	–0.10 to 0.35	Extremely broad, flattened, centered near zero

Table S4. Distribution Statistics for F1 Score (Weighted) Across Kinase Site Types.

Site Type	Median	IQR	Lower Tail	Distribution Shape
**Orthosteric (Type I/I.5/II)**	0.94–0.96	0.03–0.05	>0.90 for most	Moderately broad, right-skewed
**Type III Allosteric**	0.86–0.91	0.08–0.10	extends to ~0.75	Very broad, nearly flat
**Type IV Allosteric**	0.85–0.89	0.08–0.12	extends to <0.70	Extremely broad, collapsed toward low values

Table S5. Summary of Performance Metric Characteristics.

Metric	Common Name	Measure	Sensitivity to Class Imbalance
**AUROC**	Area Under ROC Curve	Global ranking ability	Low
**AUPR**	Area Under Precision-Recall Curve	Operational precision under imbalance	High
**MCC**	Matthews Correlation Coefficient	Balanced classification quality	Moderate
**ACC**	Accuracy	Overall correct predictions	Very low (dominated by negatives)
**F1**	F1 Score (weighted)	Harmonic mean of precision and recall	High

Table S6: Robustness Analysis of PLM Performance Metrics Across Varying Heavy-Atom Distance Thresholds.

Inhibitor Class	Contact Cutoff (Å)	Median AUROC (IQR)	Median AUPR (IQR)	Median MCC (IQR)	Median F1-Score (IQR)	Median Accuracy (IQR)
**Type I** ***(Orthosteric, Active)***	3.5 Å	0.96 (0.02)	0.74 (0.05)	0.64 (0.06)	0.66 (0.05)	0.95 (0.01)
	**4.0 Å (Baseline)**	**0.97 (0.02)**	**0.78 (0.04)**	**0.69 (0.05)**	**0.71 (0.04)**	**0.96 (0.01)**
	4.5 Å	0.96 (0.03)	0.79 (0.05)	0.67 (0.06)	0.70 (0.05)	0.95 (0.02)
	5.0 Å	0.95 (0.03)	0.77 (0.06)	0.63 (0.06)	0.67 (0.06)	0.94 (0.02)
**Type I.5** ***(Orthosteric, Trans.)***	3.5 Å	0.95 (0.02)	0.71 (0.06)	0.59 (0.07)	0.62 (0.06)	0.94 (0.02)
	**4.0 Å (Baseline)**	**0.96 (0.02)**	**0.75 (0.05)**	**0.64 (0.06)**	**0.67 (0.05)**	**0.95 (0.01)**
	4.5 Å	0.95 (0.03)	0.76 (0.05)	0.62 (0.06)	0.66 (0.05)	0.94 (0.02)
	5.0 Å	0.94 (0.04)	0.73 (0.06)	0.58 (0.07)	0.63 (0.07)	0.93 (0.02)
**Type II** ***(Orthosteric, Inactive)***	3.5 Å	0.94 (0.03)	0.66 (0.07)	0.54 (0.06)	0.58 (0.06)	0.93 (0.02)
	**4.0 Å (Baseline)**	**0.95 (0.03)**	**0.70 (0.06)**	**0.58 (0.07)**	**0.61 (0.06)**	**0.94 (0.02)**
	4.5 Å	0.94 (0.03)	0.71 (0.06)	0.57 (0.07)	0.61 (0.06)	0.93 (0.02)

	5.0 Å	0.93 (0.04)	0.69 (0.07)	0.53 (0.08)	0.58 (0.07)	0.92 (0.02)
**Type III *(Proximal Allosteric)***	3.5 Å	0.89 (0.07)	0.44 (0.11)	0.35 (0.11)	0.39 (0.10)	0.87 (0.04)
	**4.0 Å (Baseline)**	**0.91 (0.07)**	**0.49 (0.10)**	**0.41 (0.11)**	**0.45 (0.10)**	**0.90 (0.03)**
	4.5 Å	0.91 (0.08)	0.52 (0.10)	0.43 (0.12)	0.47 (0.11)	0.89 (0.04)
	5.0 Å	0.90 (0.08)	0.50 (0.11)	0.39 (0.12)	0.44 (0.11)	0.88 (0.04)
**Type IV *(Distal Allosteric)***	3.5 Å	0.66 (0.13)	0.16 (0.12)	0.11 (0.14)	0.14 (0.13)	0.83 (0.05)
	**4.0 Å (Baseline)**	**0.68 (0.13)**	**0.19 (0.13)**	**0.12 (0.14)**	**0.15 (0.13)**	**0.85 (0.05)**
	4.5 Å	0.69 (0.14)	0.21 (0.14)	0.14 (0.15)	0.17 (0.14)	0.84 (0.06)
	5.0 Å	0.68 (0.15)	0.20 (0.14)	0.12 (0.15)	0.16 (0.15)	0.82 (0.06)

Table S7: Sensitivity Analysis Evaluating the Exclusion of Highly Dynamic Segments (Activation Loop and C-Helix) on PLM Performance Metrics.

Inhibitor Class	Evaluation Regime	Median AUROC	Median AUPR	Median MCC	Median F1-Score	Median Accuracy
**Type I *(Orthosteric)***	Full-Length (Baseline)	0.97	0.78	0.69	0.71	0.96
	Loop & C-Helix Excluded	0.97	0.79	0.70	0.72	0.96
**Type I.5 *(Orthosteric)***	Full-Length (Baseline)	0.96	0.75	0.64	0.67	0.95
	Loop & C-Helix Excluded	0.95	0.74	0.63	0.65	0.95
**Type II *(Orthosteric)***	Full-Length (Baseline)	0.95	0.70	0.58	0.61	0.94
	Loop & C-Helix Excluded	0.95	0.71	0.59	0.62	0.94
**Type III *(Proximal Allosteric)***	Full-Length (Baseline)	0.91	0.49	0.41	0.45	0.90
	Loop & C-Helix Excluded	0.90	0.47	0.39	0.43	0.91
**Type IV *(Distal Allosteric)***	Full-Length (Baseline)	0.68	0.19	0.12	0.15	0.85
	Loop & C-Helix Excluded	0.68	0.18	0.11	0.14	0.86

Table S8: PLM Performance Metrics Evaluated Across Highly Granular, Homogeneous Allosteric Subtypes.

Allo Category	Granular SubPocket	Anatomical Location	Sample size	Median AUROC (IQR)	Median AUPR (IQR)	Median MCC (IQR)	Median F1-Score (IQR)
**Global Type III**	**Pooled Baseline (Table 1)**	**Proximal allosteric sites**	**30**	**0.91 (0.07)**	**0.36 (0.15)**	**0.41 (0.11)**	**0.45 (0.10)**
Type III Subtype	Subtype III-A	C-Helix Adjacent /DFG-Back Pocket	22	0.90 (0.06)	0.34 (0.12)	0.39 (0.10)	0.42 (0.09)
Type III Subtype	Other Type III Variants	Miscellaneous adjacent	8	0.93 (0.09)	0.41 (0.18)	0.44 (0.14)	0.48 (0.12)
**Global Type IV**	**Pooled Baseline (Table 1)**	**Distal allosteric sites**	**89**	**0.68 (0.13)**	**0.08 (0.04)**	**0.12 (0.14)**	**0.15 (0.13)**
Type IV Subtype	Subtype IV-A (Global)	Myristoyl Pocket (C-Lobe Distal Base)	34	0.67 (0.11)	0.10 (0.05)	0.11 (0.12)	0.13 (0.11)
Focused Target	**ABL1/ABL2 Subtype IV-A**	**C-Lobe Myristoyl subset**	**14**	**0.65 (0.08)**	**0.16 (0.07)**	**0.09 (0.08)**	**0.11 (0.08)**
Type IV Subtype	Subtype IV-B	PIF Hydrophobic Pocket (N-Lobe Surface)	55	0.69 (0.14)	0.07 (0.03)	0.14 (0.15)	0.16 (0.14)

Table S9: Distribution of Biophysical Frustration States Across PLM Classification Outcomes in ABL Kinase Structures.

PLM Classification Outcome	Binding Site Context	Targeted PDB Examples	% Minimally Frustrated	% Neutrally Frustrated	% Highly Frustrated
**True Positives (TP)**	Orthosteric ATP Cleft (Types I, I.5, II)	3K5V, 3PYY, 7N9G, 2FO0	68.5%	22.1%	9.4%
**False Negatives (FN)**	Distal Allosteric Myristoyl Pocket (Type IV)	3K5V, 3PYY, 2FO0, 1OPK	8.4%	78.9%	12.7%
**True Negatives (TN)**	Rigid Structural Core (Non-Binding Background)	All Structures	82.3%	14.2%	3.5%
**False Positives (FP)**	Flexible Surface Loops (Non-Binding Background)	All Structures	12.5%	46.0%	41.5%
